# The DNA damage-independent ATM signalling maintains CBP/DOT1L axis in *MLL* rearranged acute myeloid leukaemia

**DOI:** 10.1038/s41388-024-02998-2

**Published:** 2024-04-26

**Authors:** Guangming Wang, Wenjun Zhang, Jie Ren, Yu Zeng, Xiuyong Dang, Xiaoxue Tian, Wenlei Yu, Zheng Li, Yuting Ma, Pingping Yang, Jinyuan Lu, Junke Zheng, Bing Lu, Jun Xu, Aibin Liang

**Affiliations:** 1 Department of Hematology, Tongji Hospital, Tongji University School of Medicine, Shanghai, 200065 China; 2 East Hospital, Tongji University School of Medicine, Shanghai, 200120 China; 3 Postdoctoral Station of Clinical Medicine, Tongji Hospital, Tongji University School of Medicine, Shanghai, 200092 China; 4 Eye & ENT Hospital, Fudan University, Shanghai, 200031 China; 5 Department of Pathology, Tongji Hospital, Tongji University School of Medicine, Shanghai, 200065 China; 6 Hongqiao International Institute of Medicine, Shanghai Tongren Hospital, Key Laboratory of Cell Differentiation and Apoptosis of Chinese Ministry of Education, Faculty of Basic Medicine, Shanghai Jiao Tong University School of Medicine, Shanghai, 200025 China; 7 Shanghai Key Laboratory of Reproductive Medicine, Shanghai Jiao Tong University School of Medicine, Shanghai, 200025 China

**Keywords:** Acute myeloid leukaemia, Stress signalling, Ubiquitylation

## Abstract

The long-term maintenance of leukaemia stem cells (LSCs) is responsible for the high degree of malignancy in *MLL* (mixed-lineage leukaemia) rearranged acute myeloid leukaemia (AML). The DNA damage response (DDR) and DOT1L/H3K79me pathways are required to maintain LSCs in *MLLr*-AML, but little is known about their interplay. This study revealed that the DDR enzyme ATM regulates the maintenance of LSCs in *MLLr*-AML with a sequential protein-posttranslational-modification manner via CBP-DOT1L. We identified the phosphorylation of CBP by ATM, which confers the stability of CBP by preventing its proteasomal degradation, and characterised the acetylation of DOT1L by CBP, which mediates the high level of H3K79me2 for the expression of leukaemia genes in *MLLr*-AML. In addition, we revealed that the regulation of CBP-DOT1L axis in *MLLr*-AML by ATM was independent of DNA damage activation. Our findings provide insight into the signalling pathways involoved in *MLLr*-AML and broaden the understanding of the role of DDR enzymes beyond processing DNA damage, as well as identigying them as potent cancer targets.

## Introduction

Among the various types of leukaemia, *MLL* (mixed-lineage leukaemia) rearranged acute myeloid leukaemia (*MLLr*-AML) has a high degree of malignancy and a poor prognosis, and has been exclusively classified as one type of leukaemia since 2009 [1]. In *MLLr* leukaemia, chromosomal translocation leads to the formation of fusion genes between the *MLL* gene and its partner genes and the subsequent production of fusion proteins (MLL-fusion), such as MLL-AF6/AF9/AF10 [2]. Leukaemia stem cells (LSCs), which are responsible for the occurrence and progression of leukaemia in *MLLr*-AML, are maintained by the formation of MLL-fusion supercomplex with the SEC (super elongation complex) and DOT1L (disruptor of telomeric silencing one like) at the *HOXA* (*homeobox A*) gene locus [3, 4]. In this supercomplex, MLL-fusion, together with its interacting protein MENIN (multiple endocrine neoplasia I), are involved in chromatin localisation; the SEC is responsible for releasing paused RNA polymerase II for transcriptional elongation; and DOT1L, a methyltransferase, is responsible for the methylation of H3K79 (histone H3, lysine 79). All these players work synergistically to drive the high expression of oncogenic *HOXA* genes, such as *HOXA1/9/10* [3, 4]. As such, targeting DOT1L/H3K79me in LSCs has been shown to be a potent therapeutic approach for *MLLr-*AML [5–8].

In addition to DOT1L/H3K79me, the other characterised pathway maintaining LSCs in *MLLr-*AML is the DNA damage response (DDR), which is triggered by DNA strand breaks. Ataxia-telangiectasia mutated (ATM) and ataxia telangiectasia and Rad3 related (ATR) are critical DDR enzymes that execute cellular responses to DNA strand breaks; ATM is involved in DNA double-strand breaks (DSBs), and ATR is involved in DNA replication stress-induced DNA single-strand breaks (SSBs) [9, 10]. The evidence that these DDR enzymes support the maintenance of LSCs came from studies emphasising the critical role of ATM/ATR in the repair of DNA strand breaks, in which the inhibition of ATM or ATR kinases leads to unrepaired DNA strand breaks that trigger the myeloid differentiation of LSCs in *MLLr*-AML [11, 12].

Since both the DDR and DOT1L/H3K79me pathways are required for the maintenance of LSCs in *MLLr*-AML, interplay between each pathway should occur. Studies have shown that H3K79me is essential for binding DDR enzymes to broken genomic DNA to activate cell cycle checkpoints and repair DNA lesions [13–15]. However, the impact of DDR enzymes on the DOT1L/H3K79me pathway is largely unknown.

In this study, we investigated the role of DDR enzymes in the DOT1L/H3K79me pathway in *MLLr*-AML. We revealed that the ATM-CBP-DOT1L axis maintains murine LSCs and human AML cells with *MLL* rearrangement. The axis functions as a posttranslational protein modification cascade involving phosphorylation, acetylation, and ubiquitination, independent of DDR-related activation. Loss of *ATM* or inhibition of ATM kinase activity disrupts this axis and leads to exhaustion of LSCs and leukaemia cells in *MLLr*-AML. Our results reveal an in-depth signalling pathway in *MLLr*-AML and present a new rationale for treating cancer by targeting DDR enzymes beyond their roles in responding to DNA damage.

## Results

### *Atm* is required for the long-term maintenance of LSCs in MLL-AF9-AML mice

To investigate the role of *Atm* in the progression of *MLLr*-AML, we used an *MLL-AF9* transformed mouse model. We first transformed murine hematopoietic stem cells (Lin^-^/Sca-1^+^/c-kit^+^, LSKs) with *MLL-AF9* and an easily identified yellow fluorescent protein (YFP). Lethally irradiated wild-type C57BL/6 mice (F0 recipient) were transplanted with YFP^+^ cells, and leukaemia progression was monitored (Supplementary Fig. S1A). *MLL-AF9* transformed LSKs from either wild-type (*Atm*^*+/+*^) or *Atm* knockout (*Atm*^*−/−*^) mice survived well in vivo and induced murine AML. Although there was no significant difference between the two groups, slow leukaemic progress (YFP^+^ cells in peripheral blood) and prolonged survival were observed in *Atm*^*−/−*^ LSK-induced AML mice (Supplementary Fig. S1B, C). We also confirmed the success of AML induction by pathological examination of the liver and spleen (Supplementary Fig. S1D).

**Fig. 1:**
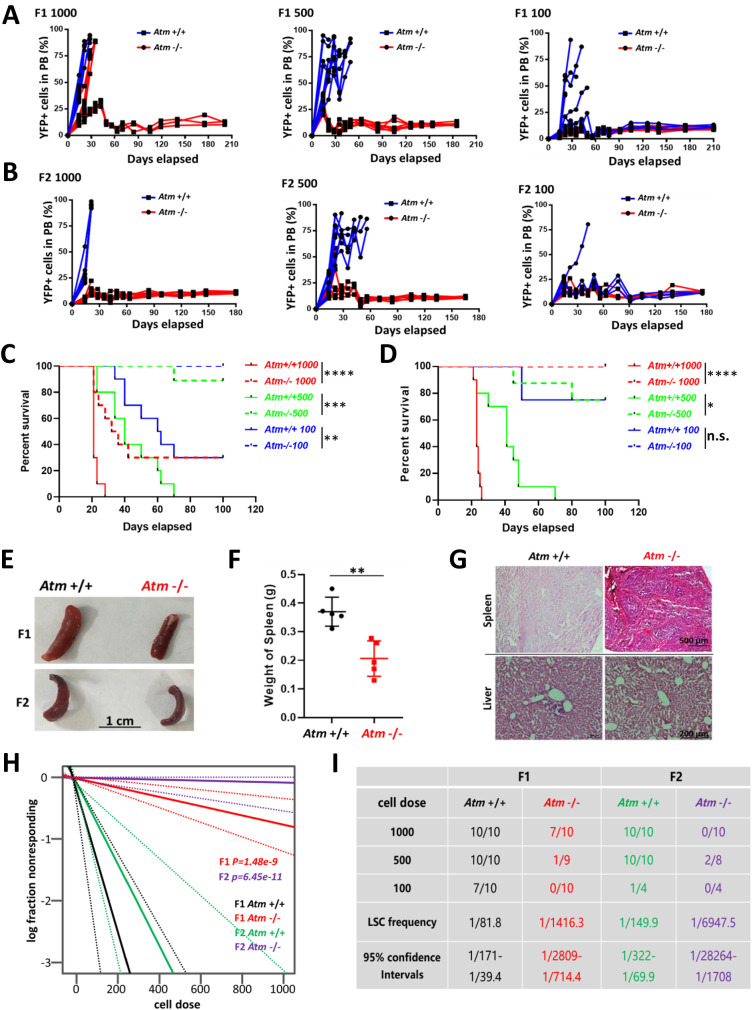
*Atm* is required for the long-term maintenance of LSCs in MLL-AF9-AML mice. YFP^+^ cells in the PB of *Atm*^+/+^ and *Atm*^−/−^ F1 (**A**) and F2 (**B**) recipient mice transplanted with 1000/500/100 LSCs were analysed (Supplementary Fig. S1E). The number of mice used for each group is shown in Fig. 1I. **P < 0.01, ***P < 0.001, ****P < 0.0001. KM survival of *Atm*^+/+^ and *Atm*^−/−^ F1 (**C**) and F2 (**D**) recipient mice transplanted with 1000/500/100 LSCs were analysed (Supplementary Fig. S1E). The number of mice used for each group is shown in Fig. 1I. **P < 0.01, ***P < 0.001, ****P < 0.0001. **E** Representative spleens of *Atm*^+/+^ and *Atm*^−/−^ F1/F2 recipient mice that received 500 LSCs at 6 weeks after transplantation are shown. **F** The spleen weights of *Atm*^+/+^ and *Atm*^−/−^ F1/F2 recipient mice that received 500 LSCs were quantified (mean ± SD) 6 weeks after transplantation. *n* = 5 mice for each group. **P < 0.01. Student’s t-test was used. **G** Representative images showing the hematoxylin-eosin staining of the liver and spleen from *Atm*^+/+^ and *Atm*^−/−^ F2 recipient mice that received 500 LSCs at 6 weeks after transplantation. **H** A logarithmic plot generated using online Extreme limiting dilution analysis (ELDA) software was generated for the fraction of nonresponding F1 and F2 recipients transplanted with different doses of LSCs. Recipients who survived until the end of survival monitoring were considered to be nonresponders. The data used for the analysis are listed in Fig. 1I. **I** The number of recipients who developed leukaemia and the total number of recipients transplanted per cell dose are listed. The frequency of LSCs in each group was calculated using online ELDA software.

We then isolated the leukaemic stem cells (LSCs, YFP^+^/ c-kit^+^) from established AML mice (F0 recipients) and employed in vivo limiting dilution transplantation assays to assess the capacity of LSCs to be affected by *Atm* loss; 1,000/500/100 LSCs from the former recipients were used to generate the next generations (Supplementary Fig. S1E). With these assays, we first found that *Atm* loss significantly impeded the progression of murine AML induced by LSCs containing *MLL-AF9*, determined by the analysis of YFP^+^ cells in the peripheral blood (Fig. 1A, B) and Kaplan-Meier survival curves of transplanted recipient mice (Fig. 1C, D). On the other hand, the splenomegaly of leukaemic mice was relieved in the *Atm*^*−/−*^ group, as evidenced by the smaller size and lower weight of the spleen compared to those in the *Atm*^*+/+*^ group (Fig. 1E, F), and leukaemia infiltration was reduced in the spleen and liver in the *Atm*^*−/−*^ group as shown by the histology analysis (Fig. 1G). Then, with online ELDA (Extreme Limiting Dilution Analysis) analysis [16], we found that the frequency of LSCs was markedly reduced in the *Atm*^*−/−*^ group compared with the *Atm*^*+/+*^ group, which was a 17-fold (1/81.8 to 1/1416.3) and 46-fold (1/149.9 to 1/6945.7) decrease in F1 and F2 recipients respectively (Fig. 1H, I). Taken together, these data demonstrate that *Atm* loss delays leukaemia progression and that *Atm* is critical for the maintenance of LSCs in the progression of AML driven by *MLL-AF9*.

### ATM deficiency downregulates the DOT1L/H3K79me pathway in MLLr-AML

**Fig. 2:**
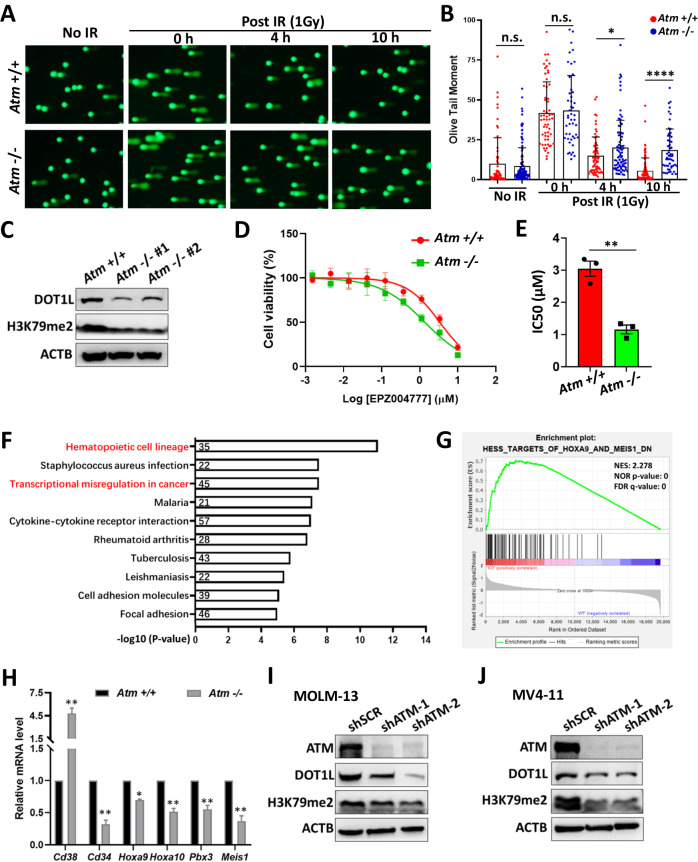
ATM deficiency downregulates the DOT1L/H3K79me pathway in MLLr-AML. **A**, **B** LSCs from *Atm*^+/+^ and *Atm*^−/−^ F2 recipients were treated with irradiation (IR, 1 Gy). The cells before (no IR) or after IR treatment were subjected to comet assay. The images represent the comet assay results showing the status of the DNA strand breaks (**A**), and the DNA strand breaks were qualified as the Olive Tail Moment (**B**). *P < 0.05, ****P < 0.0001. Student’s *t* test was used. **C** The lysates of LSCs from *Atm*^+/+^ and *Atm*^−/−^ F2 recipients were subjected to western blotting with the indicated antibodies. **D**, **E** LSCs from *Atm*^+/+^ and *Atm*^−/−^ F2 recipients were treated with different doses of the DOT1L inhibitor (EPZ004777) for 7 days. The cell viability was then determined by the CCK8 assay. Three independent experiments were performed for statistical analysis. **P < 0.01. Student’s *t* test was used. **F** LSCs from *Atm*^+/+^ and *Atm*^−/−^ F2 recipients were subjected to RNA-Seq. The genes that were differentially expressed between the two groups (Supplementary Fig. S2A) were analysed with Kyoto Encyclopedia of Genes and Genomes (KEGG) database. The top ten enriched pathways and the corresponding gene numbers are shown. **G** GSEA analysis showed that the genes with differential expression between LSCs from *Atm*^+/+^ and *Atm*^−/−^ F2 recipients were enriched in HESS_TARGETS_OF_HOXA9_AND_MEIS1_DN. **H** The relative mRNA levels of the indicated genes in LSCs from *Atm*^+/+^ and *Atm*^−/−^ F2 recipients were quantified by quantitative RT-PCR. For each gene, the mRNA expression in *Atm*^−/−^ LSCs was normalised to that in *Atm*^+/+^ LSCs. Three replicates for each gene were used for quantification (mean ± SD). *P < 0.05, **P < 0.01. Student’s *t* test was used. **I**, **J** The cells were transduced with lentivirus expressing control shRNA (shSCR) or shRNA targeting *ATM* (shATM) and puromycin acetyltransferase for selection. The transduced cells were selected with 1 μg/ml puromycin for at least three days, after which the total lysates of the control (shSCR) and *ATM* knockdown (shATM) MOLM-13 (**I**) and MV4-11 (**J**) cells were subjected to western blotting with the indicated antibodies.

Since it has been reported that the inhibition of ATM kinase compromises DNA repair and leads to accumulated DNA damage [11], we first examined how *Atm* loss affects the DNA damage and repair of mouse LSCs. With the comet assay, we found that, while there were comparable amounts of DNA strand breaks in *Atm*^*+/+*^ and *Atm*^*−/−*^ LSCs from F2 recipients, the *Atm*^*−/−*^ LSCs exhibited less efficient repair of DNA strand breaks when challenged with irradiation (IR) (Fig. 2A, B). These results demonstrated that although ATM plays an essential role in repairing exogenously induced DNA damage, its loss may not necessarily lead to the physiological accumulation of DNA strand breaks in mouse *MLLr*-AML LSCs.

We then focused on our primary goal to investigate the role of ATM in the DOT1L/H3K79me pathway. We checked whether DOT1L and H3K79me were affected by *Atm* loss, and surprisingly, we found that the protein level of DOT1L and the H3K79me2 were markedly reduced in *Atm*^*−/−*^ LSCs (Fig. 2C). Furthermore, the *Atm*^*−/−*^ LSCs were more sensitive to the DOT1L inhibitor than *Atm*^*+/+*^ LSCs were (Fig. 2D, E). We also performed a transcriptome profiling with *Atm*^*+/+*^ and *Atm*^*−/−*^ LSCs isolated from F2 recipients to identify the transcriptional changes affected by *Atm* loss. KEGG (Kyoto Encyclopedia of Genes and Genomes) analysis revealed that genes differentially expressed between *Atm*^*+/+*^ and *Atm*^*−/−*^ LSCs were correlated with the hematopoietic cell lineage and transcriptional misregulation in cancer (Fig. 2F and Supplementary Fig. S2A). With GSEA (gene set enrichment analysis), we found that the genes supporting myeloid differentiation and genes negatively regulated by HOXA9 and MEIS1 were upregulated in *Atm*^*−/−*^ LSCs (Fig. 2G and Supplementary Fig. S2B) [17–20]. A heatmap of gene expression and quantitative RT-PCR results confirmed that hematopoietic stem cell markers (e.g., *Cd34*) and several pivotal LSC maintenance elements (e.g., *Hoxa10, Pbx3 and Meis1*) were downregulated in *Atm*^*−/−*^ LSCs (Fig. 2H and Supplementary Fig. S2C, D).

To investigate the role of *ATM* in human *MLLr*-AML, we knocked down *ATM* in *MLL-AF9* (MOLM-13) and *MLL-AF4* (MV4-11) leukaemia cells via a lentivirus-based shRNA expression technique. We used lentiviruses simultaneously expressing shRNA and green fluorescent protein (GFP) to monitor the growth of cells affected by *ATM* knockdown. The proportion of GFP^+^ cells was consistent during culture for the control (shSCR) cells, but decreased sharply for the *ATM*-knockdown cells (Supplementary Fig. S2E–H), suggesting that effective *ATM* knockdown blocked the growth of the MOLM-13 and MV4-11 cells. We verified that the DOT1L protein level was significantly reduced in *ATM*-knockdown cells, and this change was accompanied by a decrease in H3K79me2 levels in MV4-11 and MOLM-13 (Fig. 2I, J). In contrast, no change in the mRNA level of *DOT1L* was found in mouse *Atm*^*−/−*^ LSCs or in human *ATM* knockdown *MLLr* leukaemia cells (Supplementary Fig. S2I, J).

These results explain the functional deficiency of *Atm*^*−/−*^ LSCs in the progression of leukaemia and indicate that *Atm* may support the maintenance of LSCs through the regulation of DOT1L and H3K79me, as they are the predominant upstream targets of HOXA, MEIS1 and PBX3 [3, 4]. We then proposed that ATM regulates DOT1L at the protein level, contributing to the long-term maintenance of LSCs and the survival of *MLLr*-AML cells.

### ATM protects the DOT1L protein from ubiquitination-mediated degradation

**Fig. 3:**
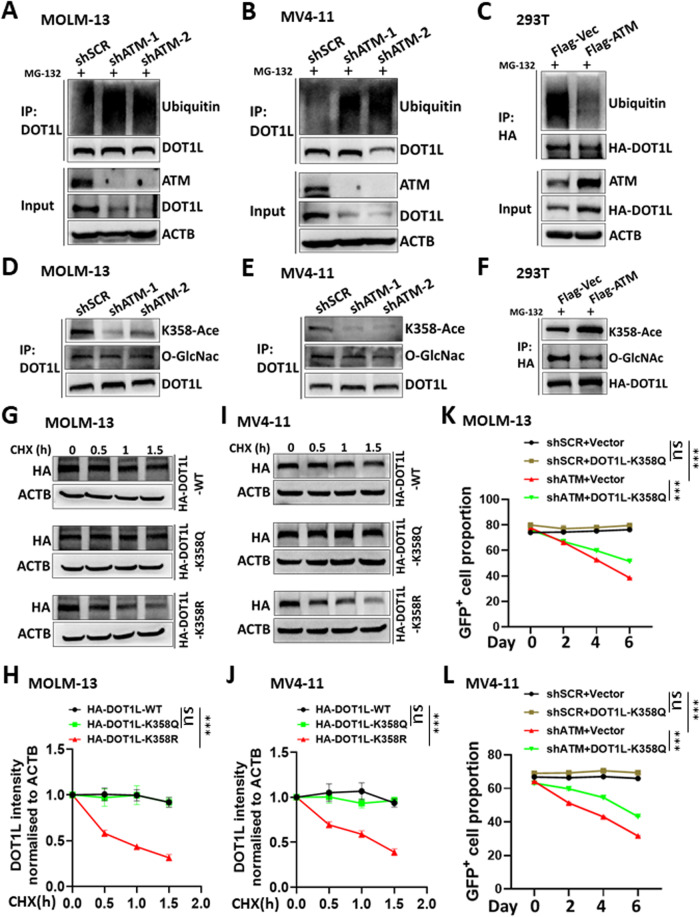
ATM protects the DOT1L protein from ubiquitination-mediated degradation. Control (shSCR) and *ATM* knockdown (shATM) MOLM-13 (**A**) and MV4-11 (**B**) cells were incubated with 25 μM MG132 for 4 h. The cell lysates were subjected to immunoprecipitation (IP) with DOT1L antibodies. The input and IP samples were then detected by western blotting with the indicated antibodies. **C** 293T cells were cotransfected with HA-DOT1L and control vector (Flag-Vec) or Flag-ATM. At 36 h posttransfection, the cells were treated with 25 μM MG132 for 20 h. The cell lysates were subjected to immunoprecipitation (IP) with HA antibodies. The input and IP samples were then detected by western blotting with the indicated antibodies. The IP samples of MOLM-13 (**D**), MV4-11 (**E**), and 293T (**F**) cells were detected by western blotting with the indicated antibodies. MOLM-13 (**G**, **H**) and MV4-11 (**I**, **J**) cells were transduced with lentivirus expressing wild-type (WT) or mutant HA-DOT1L (K358Q, K358R). The cells were incubated with 40 μg/ml CHX for the indicated times. The total lysates of the cells were then subjected to western blotting with the indicated antibodies. The band intensity of HA was normalised to that of ACTB of the same sample, and each time point from three independent experiments was normalised to time 0 for statistical analysis. The difference in slopes between every two groups was compared. ns, not significant. ***P < 0.001. MOLM-13 (**K**) and MV4-11 (**L**) cells were transduced with lentivirus expressing HA-DOT1L (K358Q) or not (vector). The cells were then transduced with lentivirus expressing shRNA and green fluorescent protein (GFP). The cells were subjected to flow cytometry (see Supplementary Fig. S1C for gating) to analyse the proportion of GFP^+^ cell. The analysis was performed every two days during the culture. Three replicates for each time point for different cells were used for linear regression analysis. The difference in slopes between every two groups was compared. ns not significant. ***P < 0.001. Three independent experiments were performed.

The downregulated DOT1L protein might result from protein degradation, and we observed apparent DOT1L degradation in MOLM-13 and MV4-11 cells treated with cycloheximide (CHX), a protein synthesis inhibitor (Supplementary Fig. S3A). To determine how DOT1L was degraded, we challenged cells with MG132, a proteasome inhibitor, or chloroquine (CHQ), a lysosomal inhibitor. We found that only MG132 could prevent DOT1L degradation, which indicated that mainly proteasome-mediated DOT1L degradation occurred in MV4-11 and MOLM-13 cells (Supplementary Fig. S3B, C). As protein ubiquitination occurs before degradation by the proteasome, we next examined the extent to which ubiquitination of DOT1L was affected by ATM. Indeed, the ubiquitination of the DOT1L protein was significantly elevated when *ATM* was knocked down in both MOLM-13 and MV4-11 cells (Fig. 3A, B). After demonstrating the loss-of-function effect of ATM on DOT1L, we sought to validate the role of ATM in DOT1L through gain-of-function experiments. ATM is encoded by an mRNA sequence containing approximately 13k base pairs, which hinders the overexpression of *ATM* in leukaemia cells but is feasible in 293T cells. In 293T cells, we also found that downregulated ATM led to reduced DOT1L protein levels in combination with increased ubiquitination of DOT1L (Supplementary Fig. S3D, E). In contrast, the overexpression of *ATM* upregulated DOT1L protein levels and decreased its ubiquitination (Supplementary Fig. S3F and Fig. 3C).

Next, we aimed to decipher the relationship between DOT1L and ATM. ATM is thought to phosphorylate DOT1L as a protein kinase, but the phosphorylation of DOT1L has rarely been reported by either biochemical assay or massive proteomics [21, 22]. With the coimmunoprecipitation (co-IP) assay, we did not observe interactions between DOT1L and ATM endogenously or exogenously (Supplementary Fig. S3G–J). Thus, we speculated that the regulation of DOT1L protein stability by ATM might be indirect.

Two recent studies demonstrated that the acetylation and O-GlcNAcylation of DOT1L promote its stability [23, 24]. Therefore, we checked whether the acetylation and O-GlcNAcylation of DOT1L are affected by ATM. While O-GlcNAcylation was unaffected, the acetylation of DOT1L K358 was downregulated in *ATM* knockdown MOLM-13 and MV4-11 cells (Fig. 3D, E). Furthermore, the overexpression of *ATM* upregulated the acetylation of DOT1L K358 in 293T cells (Fig. 3F). To further confirm the relationship between the acetylation of DOT1L K358 and its degradation, we generated mutant DOT1L with K358Q to mimic constitutive acetylation and K358R to prevent acetylation. When challenged with CHX, DOT1L (K358R) was degraded more rapidly, although little difference was observed between DOT1L (WT) and DOT1L (K358Q) in MOLM-13 and MV4-11 cells (Fig. 3G–J). Notably, the inhibition of MOLM-13 and MV4-11 cell growth caused by *ATM* knockdown was attenuated by the overexpression of DOT1L (K358Q) (Fig. 3K, L and Supplementary Fig. S3K, L).

### CBP-mediated DOT1L-K358 acetylation confers DOT1L stability in *MLLr*-AML

**Fig. 4:**
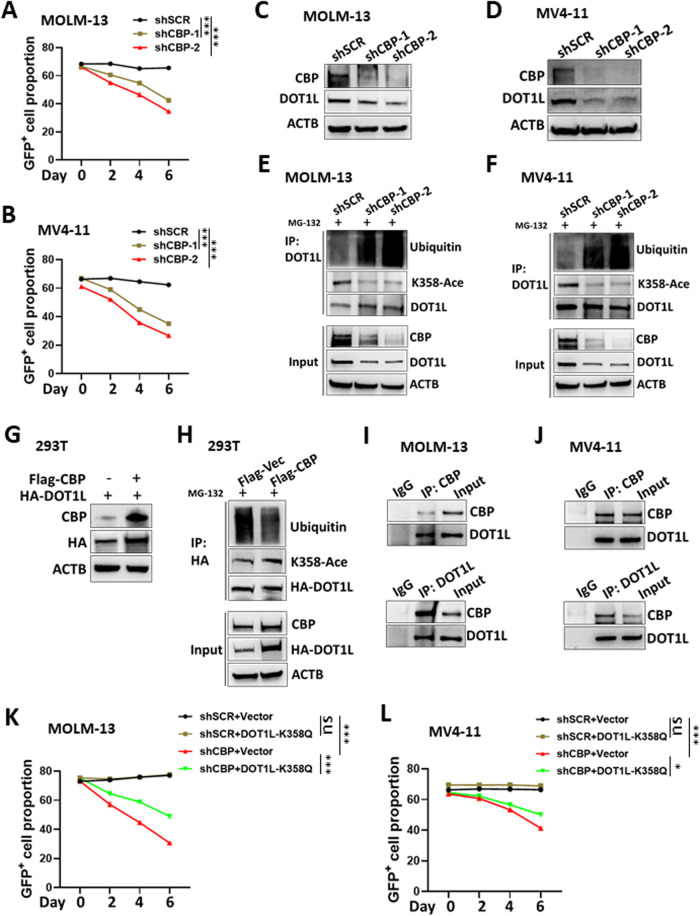
CBP-mediated DOT1L-K358 acetylation confers DOT1L stability in *MLLr*-AML. **A**, **B** The cells were transduced with lentivirus expressing control shRNA (shSCR) or shRNA targeting *CBP* (shATM) along with GFP. The cells were subjected to flow cytometry (same gating strategy as in Supplementary Fig. S1C) to analyse the proportion of GFP^+^ cells among the MOLM-13 (**A**) and MV4-11 (**B**) cells. The analysis was performed every two days during the culture. Three replicates for each time point for different cells were used for linear regression analysis. The difference in slopes between every two groups was compared. ***P < 0.001. Three independent experiments were performed. The total lysates of the control (shSCR) and *CBP* knockdown (shCBP) MOLM-13 (**C**) and MV4-11 (**D**) cells were subjected to western blotting with the indicated antibodies. Control (shSCR) and *CBP* knockdown (shCBP) MOLM-13 (**E**) and MV4-11 (**F**) cells were incubated with 25 μM MG132 for 4 h. The cell lysates were subjected to immunoprecipitation (IP) with DOT1L antibodies. The input and IP samples were then detected by western blotting with the indicated antibodies. **G** HA-DOT1L with or without Flag-CBP were cotransfected into 293T cells. The cell lysates were then subjected to western blotting with the indicated antibodies. **H** 293T cells were cotransfected with HA-DOT1L and a control vector (Flag-Vec) or Flag-CBP. At 36 h posttransfection, the cells were treated with 25 μM MG132 for 20 h. The cell lysates were subjected to immunoprecipitation (IP) with HA antibodies. The input and IP samples were then detected by western blotting with the indicated antibodies. **I**, **J** MOLM-13 (**G**) and MV4-11 (**H**) cell lysates were subjected to IP with CBP, DOT1L, or control IgG. The input and IP samples were then detected by western blotting with the indicated antibodies. MOLM-13 (**K**) and MV4-11 (**L**) cells were transduced with lentivirus expressing HA-DOT1L (K358Q) or not (vector). The cells were then transduced with lentivirus expressing shRNA along with green fluorescent protein (GFP). The cells were subjected to flow cytometry (See Supplementary Fig. S1C for gating) to analyse the proportion of GFP^+^ cells. The analysis was performed every two days during the culture. Three replicates for each time point for different cells were used for linear regression analysis. The difference in slopes between every two groups was compared. ns, not significant. *P < 0.05, ***P < 0.001. Three independent experiments were performed.

After demonstrating the function of DOT1L-K358 acetylation in *MLLr*-AML, we focused on CBP, a protein acetyltransferase reported to be responsible for DOT1L-K358 acetylation [23]. With the lentivirus-based shRNA expression technique, we found that efficient *CBP* knockdown inhibited cell growth (Fig. 4A, B and Supplementary Fig. S4A, B) and downregulated the DOT1L protein (Fig. 4C, D). However, the mRNA level of *DOT1L* was undisturbed in MOLM-13 and MV4-11 cells (Supplementary Fig. S4C, D). Furthermore, the acetylation of DOT1L-K358 was decreased, while the ubiquitination of DOT1L was increased in *CBP* knockdown MOLM-13 and MV4-11 cells (Fig. 4E, F). Similar results were obtained with 293T cells under *CBP* knockdown (Supplementary Fig. S4E, F). On the other hand, the overexpression of CBP upregulated the expression of exogenously expressed DOT1L protein, which was probably achieved by the upregulating acetylation and downregulating ubiquitination of DOT1L in 293T cells (Fig. 4G, H). The endogenous CBP and DOT1L proteins were coimmunoprecipitated with each other in MOLM-13 and MV4-11 cells (Fig. 4I, J). In addition, the inhibition of MOLM-13 and MV4-11 cell growth caused by *CBP* knockdown was alleviated by the overexpression of DOT1L(K358Q) (Fig. 4K, L and Supplementary Fig. S4G, H). These results indicated that CBP directly mediated the K358 acetylation of DOT1L to confer stability in *MLLr*-AML cells.

### ATM confers CBP stability by preventing its ubiquitination-mediated degradation

**Fig. 5:**
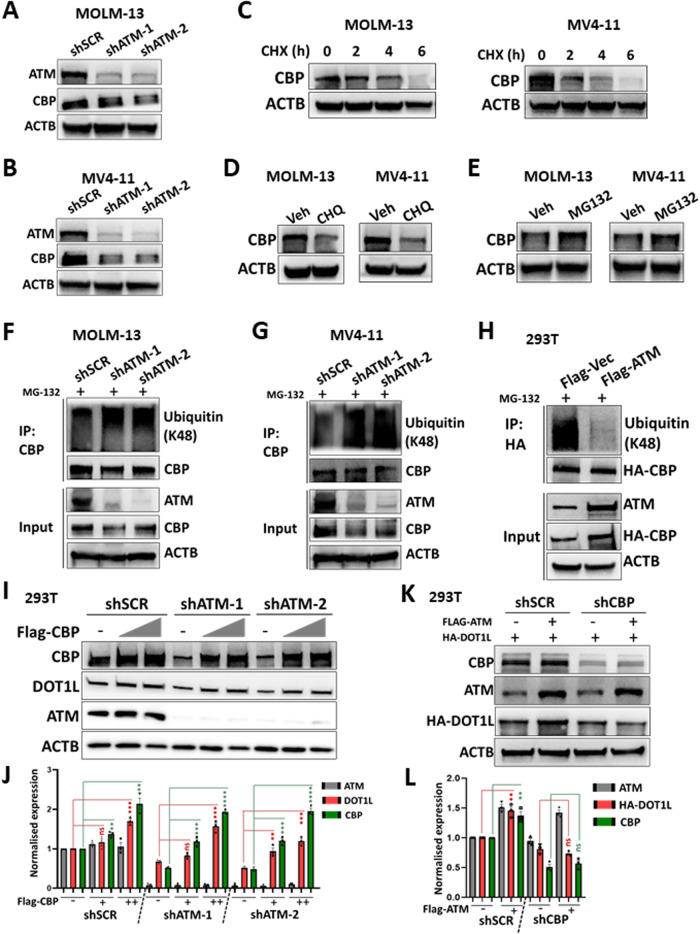
ATM confers CBP stability by preventing its ubiquitination-mediated degradation. The total lysates of control (shSCR) and *ATM* knockdown (shATM) MOLM-13 (**A**) and MV4-11 (**B**) cells were subjected to western blotting with the indicated antibodies. **C** MOLM-13 and MV4-11 cells were incubated with 40 μg/ml CHX for the indicated times. The total lysates of the cells were then subjected to western blotting with the indicated antibodies. **D** MOLM-13 and MV4-11 cells were incubated with DMSO (vehicle, Veh) or 50 μM chloroquine (CHQ) for 24 h. The total lysates of the cells were then subjected to western blotting with the indicated antibodies. **E** MOLM-13 and MV4-11 cells were incubated with 40 μg/ml CHX in combination with DMSO (vehicle, Veh) or 25 μM MG132 for 6 h. The total lysates of the cells were then subjected to western blotting with the indicated antibodies. Control (shSCR) and *ATM* knockdown (shATM) MOLM-13 (**F**) and MV4-11 (**G**) cells were treated with 25 μM MG132 for 4 h. The cell lysates were subjected to IP with CBP antibodies. The input and IP samples were then detected by western blotting with the indicated antibodies. **H** 293T cells were cotransfected with HA-CBP and control vector (Flag-Vec) or Flag-ATM. At 36 h posttransfection, the cells were treated with 25 μM MG132 for 20 h. The cell lysates were subjected to immunoprecipitation (IP) with HA antibodies. The input and IP samples were then detected by western blotting with the indicated antibodies. **I**, **J** Flag-CBP was transfected into control (shSCR) or *ATM* knockdown (shATM) 293T cells. After 72 h, the cells were collected, and the cell lysates were subjected to western blotting with the indicated antibodies (**I**). The band intensity of ATM/DOT1L/CBP was normalised to that of ACTB in the same sample, and the data from three independent experiments were used for statistical analysis (**J**). Student’s *t* test was performed for significant differences. ns not significant. **P < 0.01, ***P < 0.001, ****P < 0.0001. **K**, **L** HA-DOT1L with or without Flag-ATM were cotransfected into control (shSCR) or *CBP* knockdown (shCBP) 293T cells. After 72 h, the cells were collected, and the cell lysates were subjected to western blotting with the indicated antibodies (**K**). The band intensity of ATM/HA-DOT1L/CBP was normalised to that of ACTB in the same sample, and the data from three independent experiments were used for statistical analysis (**L**). Student’s *t* test was performed for significant differences. ns not significant. **P < 0.01, ***P < 0.001, ****P < 0.0001.

To determine the impact of ATM on CBP, we first checked the mRNA and protein levels of *CBP* following *ATM* knockdown. We found that *ATM* knockdown led to a decrease in the protein level of CBP, but no change in the mRNA level was observed in MOLM-13 or MV4-11 cells (Fig. 5A, B and Supplementary Fig. S5A–D), implying that ATM might also ensure CBP protein stability. We then treated MOLM-13 and MV4-11 cells with CHX, CHQ or MG132 to test the degradation of CBP as previously described for DOT1L protein stability. Like that of the DOT1L protein, degradation of CBP was evident in both MOLM-13 and MV4-11 cells (Fig. 5C). The degradation of CBP was mediated mainly by the proteasome since MG132, not CHQ, increased the protein level of CBP (Fig. 5D, E).

Polyubiquitin chains can be linked by different lysine residues (K6, K11, K27, K29, K33, K48, and K63) within ubiquitin, and K48-linked polyubiquitin targets proteins for proteasomal degradation [25]. CBP could be polyubiquitinated mainly by K6-, K48- and K63-linked ubiquitin in 293T cells (Supplementary Fig. S5E). Thus, the specific K48-linked ubiquitination of CBP indicates its degradation. As expected, the K48-linked ubiquitination of the CBP protein was significantly elevated following *ATM* knockdown (Fig. 5F, G). As the reduction in ATM decreased CBP protein levels through ubiquitination-mediated degradation, we wondered whether the increase in ATM expression would reverse this effect and verified this finding in 293T cells. While *ATM* knockdown decreased the protein level and increased the K48-linked ubiquitination of CBP in 293T cells (Supplementary Fig. S5F, G), the overexpression of ATM significantly improved the CBP protein level by decreasing K48-linked ubiquitination (Fig. 5H and Supplementary Fig. S5H). These data confirmed that ATM confers CBP protein stability by preventing its ubiquitination-mediated degradation.

Since we previously demonstrated that DOT1L serves as a downstream target of both ATM and CBP, we identified CBP as a candidate for ATM treatment. Therefore, it was reasonable to speculate that CBP could act as an intermediate between ATM and DOT1L. To test to this hypothesis, we first overexpressed CBP following *ATM* knockdown and found that the reduction in the DOT1L protein caused by *ATM* knockdown was reversed by the overexpression of CBP (Fig. 5I, J). In contrast, the molecular signature associated with elevated DOT1L protein expression in the context of *ATM* overexpression was abolished by *CBP* knockdown (Fig. 5K, L). These results revealed that CBP serves as an intermediate between ATM and DOT1L.

### S1578 phosphorylation mediated by ATM stabilizes CBP

**Fig. 6:**
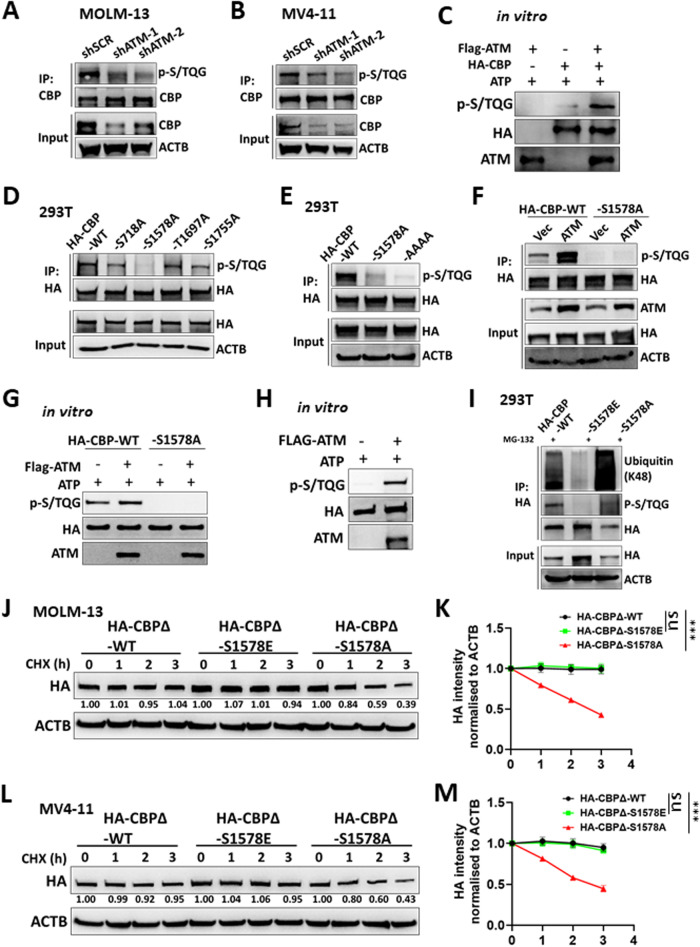
S1578 phosphorylation mediated by ATM stabilised CBP. Control (shSCR) and *ATM* knockdown (shATM) MOLM-13 (**A**) and MV4-11 (**B**) cells were treated with 25 μM MG132 for 4 h. The cell lysates were subjected to IP with CBP antibodies. The input and IP samples were then detected by western blotting with the indicated antibodies. **C** 293T cells were transfected with HA-CBP or Flag-ATM, and HA-CBP or Flag-ATM was purified. Purified HA-CBP was subjected to in vitro phosphorylation with or without Flag-ATM, and the reactions were then detected by western blotting with the indicated antibodies. **D** Wild-type (WT) CBP and different HA-tagged CBP (HA-CBP) mutants (S718A, S1578A, T1697A, and S1755A) were transfected into 293T cells. After 72 h, the cell lysates were subjected to IP with HA antibodies. The input and IP samples were then detected by western blotting with the indicated antibodies. **E** The WT, S1578A and AAAA (S718A/S1578A/T1697A/S1755A) HA-CBP were transfected into 293T cells. After 72 h, the cell lysates were subjected to IP with HA antibodies. The input and IP samples were then detected by western blotting with the indicated antibodies. **F** WT and S1578A HA-CBP with or without Flag-ATM were transfected into 293T cells. 72 h later, the cell lysates were subjected to IP with HA antibodies. The Input and IP samples were then detected by western blotting with the indicated antibodies. **G** 293T cells were transfected with WT or S1578A HA-CBP, Flag-ATM. HA-CBP or Flag-ATM was purified. Purified HA-CBP was subjected to in vitro phosphorylation with or without Flag-ATM, and the reactions were then detected by western blotting with the indicated antibodies. **H** Purified HA-CBP was subjected to in vitro dephosphorylation with Lambda PP at 30 °C for 3 h. Dephosphorylated HA-CBP was then subjected to in vitro phosphorylation with or without Flag-ATM, and the reactions were detected by western blotting with the indicated antibodies. **I** WT, S1578A, and S1578E HA-CBP were transfected into 293T cells. At 36 h posttransfection, the cells were treated with 25 μM MG132 for 20 h. The cell lysates were subjected to IP with HA antibodies. The input and IP samples were then detected by western blotting with the indicated antibodies. MOLM-13 (**J**, **K**) and MV4-11 (**L**, **M**) cells were transduced with lentivirus expressing wild-type (WT) or mutant HA-CBPΔ (S1578E, S1578A). The cells were incubated with 40 μg/ml CHX for the indicated times. The total lysates of the cells were then subjected to western blotting with the indicated antibodies. The band intensity of HA was normalised to that of ACTB in the same sample, and each time point from three independent experiments was normalised to time 0 for statistical analysis. The difference in slopes between every two groups was compared. ns not significant. ***P < 0.001.

We next sought to determine the regulatory effect of ATM on CBP. With a specific antibody targeting the phosphorylated ATM/ATR substrate motif (p-S/TQG), we found that *ATM* knockdown led to a decrease in the phosphorylated S/TQG motif of CBP in both MOLM-13 and MV4-11 cells (Fig. 6A, B). Furthermore, while we evaluated the interaction between ATM and CBP, the overexpression of ATM increased the phosphorylation of the S/TQG motif of CBP in vivo (Supplementary Fig. S6A–C). Notably, when ectopically expressed CBP was purified, ATM increased the phosphorylation of CBP S/TQG motif in vitro (Fig. 6C). Taken together, these results indicate that ATM phosphorylates CBP.

We further aimed to identify the specific sites in CBP phosphorylated by ATM. The CBP protein has four conserved S/TQG sites (S718, S1578, T1697 and S1755) across humans, monkeys, mice, rats, pigs, bovines, horses, rabbits and chickens (Supplementary Fig. S6D). We generated various CBP mutants by substituting serine (S) with alanine (A) to block phosphorylation. When a single site was mutated, we found that only S1578A significantly reduced the S/TQG motif-mediated phosphorylation of CBP (Fig. 6D). The S/TQG motif phosphorylation of the S1578A mutant was comparable to that of the S1578A double site mutants (S1578A/S718A, S1578A/T1697A, and S1578A/S1755A) (Supplementary Fig. S6E). Furthermore, the S1578A mutation decreased the S/TQG motif-mediated phosphorylation of CBP to a level comparable to that of the AAAA (S718A/ S1578A/ T1697A/S1755A) mutant (Fig. 6E). In contrast, the S718A/T1697A/S1755A mutation had little impact on the S/TQG motif phosphorylation of CBP (Supplementary Fig. S6F). These results indicate that S1578 is the dominant phosphorylation site of the S/TQG motif in CBP.

We next wanted to determine whether S1578 is the site at which ATM phosphorylates CBP. With in vivo and in vitro assays, we found that, while increasing S/TQG motif phosphorylation in the wild-type (WT) CBP, ATM did not play the same role in the S1578A mutant in vivo or in vitro (Fig. 6F, G). These results suggested the necessity of S1578 for CBP phosphorylation by ATM. Nevertheless, they could not define S1578 as the site of CBP phosphorylation by ATM since we cannot exclude the possibility that the basal level of S/TQG motif phosphorylation in CBP is phosphorylated S1578 by other kinases, which is required for subsequent CBP phosphorylation by ATM. As such, we used a protein phosphatase to eliminate the phosphorylated residues in CBP (Supplementary Fig. S6G). We then performed in vitro phosphorylation assays and found that ATM phosphorylated dephosphorylated CBP (Fig. 6H). Taken together, these results reveal that ATM phosphorylates S1578 in CBP.

To confirm the relationship between the phosphorylation of CBP S1578 and its degradation, we generated a mutant CBP construct in which S1578E mimicked constitutive phosphorylation. We found that, compared with that in CBP (WT), K48-linked ubiquitination was increased in CBP (S1578A) but was decreased in CBP (S1578E) in 293T cells (Fig. 6I). We also investigated the phosphorylation-related degradation of CBP in *MLLr*-AML cells. Since the long mRNA sequence of *CBP* (~7.5 k base pairs) hinders the stable expression of mutant CBP in *MLLr*-AML cells via lentivirus-based techniques, we generated a truncated CBP lacking amino acids 1-1092 (CBPΔ) compared to the full-length CBP (Supplementary Fig. S6H). When CBPΔ was coexpressed in 293T cells, the DOT1L protein level increased, and the level was increased by ATM (Supplementary Fig. S6I–K), indicating that the properties of CBPΔ were similar to those of full-length CBP. We subsequently expressed the mutant CBPΔ in *MLLr*-AML and 293T cells and found that the S1578A CBPΔ exhibited significantly more rapid degradation than did the WT and S1578E CBPΔ (Fig. 6J–M and Supplementary Fig. S6L). These results demonstrated that the phosphorylation of CBP S1578 prevents its degradation.

### The kinase activity of ATM is essential for regulating CBP but is DNA damage independent

**Fig. 7:**
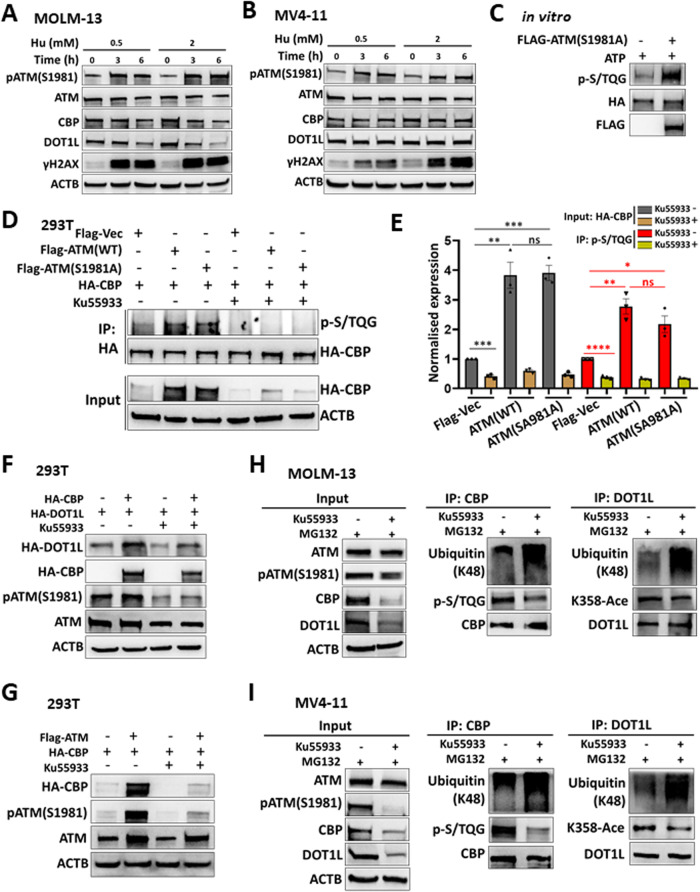
The regulatory effect of ATM on CBP and DOT1L is DNA damage independent. MOLM-13 (**A**) and MV4-11 (**B**) cells were treated with hydroxyurea (Hu, 0.5 mM or 2 mM) for the indicated times. The lysates were extracted and detected by western blotting with the indicated antibodies. **C** The purified HA-CBP was subjected to in vitro phosphorylation with or without Flag-ATM(S1981A), and the reactions were then detected by western blotting with the indicated antibodies. **D**, **E** HA-CBP was cotransfected with FLAG-Vec, FLAG-ATM (WT), or FLAG-ATM(S1981A) into 293T cells. After 24 h, the cells were treated with or without 25 μM Ku55933 for 48 h. The cells were collected, and the cell lysates were then subjected to IP with HA antibodies. The input and IP samples were then detected by western blotting with the indicated antibodies (**D**). For the input samples, the band intensity of HA was normalised to that of ACTB in the same sample, and that of p-S/TQG was normalised to that of HA for the IP samples. The data from three independent experiments were used for statistical analysis (**E**). Student’s *t* test was performed for significant differences. ns not significant. *P < 0.05, **P < 0.01, ***P < 0.001, ****P < 0.0001. **F**. HA-DOT1L was cotransfected with or without HA-CBP into 293T cells. After 24 h, the cells were treated with or without 25 μM Ku55933 for 48 h. The cells were collected, and the cell lysates were then subjected to western blotting with the indicated antibodies. **G**. HA-CBP was cotransfected with or without FLAG-ATM into 293T cells. After 24 h, the cells were treated with or without 25 μM Ku55933 for 48 h. The cells were collected, and the cell lysates were then subjected to western blotting with the indicated antibodies. MOLM-13 (**H**) and MV4-11 (**I**) cells were treated with 25 μM Ku55933 for 20 h and 25 μM MG132 for 4 h. The cell lysates were then subjected to IP with CBP or DOT1L antibodies. The input and IP samples were then detected by western blotting with the indicated antibodies.

The DNA damage-activated phosphorylation of ATM(S1981) has been proven to be essential for the subsequent phosphorylation of substrates during the DNA damage response [26, 27]. We then investigated the role of the phosphorylation of ATM (S1981) in maintaining the stability of the CBP protein. When MOLM-13 and MV4-11 cells were treated with hydroxyurea (Hu) or etoposide (ETO), the phosphorylation of ATM(S1981) and H2A.X variant histone (γH2AX) was elevated; however, the protein levels of CBP and DOT1L were not upregulated, as expected; rather, they were downregulated by a higher dose or longer duration of treatment (Fig. 7A, B and Supplementary Fig. S7A–F). In 293T cells, the overexpressed ATM (S1981A) mutant could upregulate the CBP protein to the same extent as the wild-type ATM (Supplementary Fig. S7G). In addition, the ATM (S1981A) mutant could phosphorylate CBP in vitro and increase CBP phosphorylation in vivo (Fig. 7C–E). These results demonstrated that the phosphorylation of ATM(S1981) is not responsible for the phosphorylation or the stability of the CBP.

To validate the role of the kinase activity of ATM in CBP phosphorylation, we employed the ATM kinase-specific inhibitor, Ku55933. In 293T cells, treatment with Ku55933 downregulated the expression of the CBP protein and the S/TQG phosphorylation of CBP (Fig. 7D, E). Notably, the upregulation of DOT1L by CBP and that of CBP by ATM was attenuated by Ku55933 treatment (Fig. 7F, G). While Ku55933 blocked the phosphorylation of ATM, the protein levels of CBP and DOT1L were significantly decreased upon Ku55933 treatment in human *MLLr*-AML cells (Supplementary Fig. S7H, I). Furthermore, the downregulation of the CBP and DOT1L proteins upon Ku55933 treatment was accompanied by decreased CBP S/TQG motif phosphorylation and DOT1L-K358 acetylation but increased K48-linked polyubiquitination (Fig. 7H, I). Collectively, these results demonstrated that the regulation of CBP by ATM was dependent on the kinase activity of ATM.

## Discussion

The present study revealed that the ATM-CBP-DOT1L axis regulates the maintenance of *MLLr*-AML LSCs and leukaemia cells through sequential protein posttranslational modifications. ATM phosphorylates CBP(S1578) to confer its stability by preventing ubiquitination-mediated proteasomal degradation in an ATM (S1981)-independent manner. The stabilised CBP acetylates DOT1L(K358) to protect it from proteasomal degradation, mediating the high level of H3K79me2 for the upregulated expression of leukaemia genes in *MLLr*-AML.

### Degradation of the DOT1L protein in *MLLr*-AML

DOT1L and H3K79 methylation are critical players in *MLLr* leukaemia. The methylation of H3K79, as one of the most prominent histone modifications, mediates the transcriptional activation of *MLLr-*related oncogenes, which serves as a necessary guarantee to drive the occurrence of leukaemia and to maintain the self-renewal ability of LSCs in *MLLr* leukaemia [5, 28]. DOT1L, the only methyltransferase for H3K79 in mammals known to date, methylates H3K79 to activate the expression of *HOXA* and *MEIS1* in LSCs to support the occurrence of leukaemia and to maintain the growth of *MLLr* leukaemia cells [29–32]. As such, the inhibitors targeting DOT1L have been developed and tested in clinical trials, and the development of improved DOT1L inhibitors has been ongoing since 2013 [6, 8, 33, 34]. In our study, the DOT1L protein and related H3K79 methylation were downregulated by *ATM* deficiency in *MLLr*-AML, and the cellular and molecular signatures affected by *ATM* deficiency resembled those caused by a lack of *DOT1L*; as such, we primarily considered DOT1L to be a downstream target of ATM that regulates LSC maintenance and *MLLr*-AML cell survival.

The downregulated DOT1L protein expression in response to *ATM* loss is attributed to upregulated ubiquitination-mediated proteasomal degradation in *MLLr*-AML cells. According to recent studies, the stability of the DOT1L protein is conferred by several posttranslational modifications, such as O-GlcNAcylation and acetylation, which prevent DOT1L from binding to E3 ubiquitin ligases and subsequent proteasome degradation, respectively, in *MLLr* leukaemia cells and colorectal cancer cells [23, 24]. We observed that the change in the level of acetylation rather than the O-GlcNAcylation of DOT1L was affected by ATM. Like in colorectal cancer cells, we confirmed that the acetylation of DOT1L-K358 is responsible for its stability in *MLLr* leukaemia cells. On the other hand, mutated DOT1L mimicking constitutive acetylation (K358Q) can rescue the growth deficiency of *MLLr* leukaemia cells with *ATM* loss, which validates our speculation that DOT1L serves as a downstream target of ATM.

### The role of CBP and its regulation in *MLLr* leukaemia

Our study has revealed that CREB binding protein (CBP) is a linker between ATM and DOT1L that sustains *MLLr* leukaemia. CBP is an acetyltransferase for histones and nonhistone proteins and plays critical roles in normal development and abnormal malignancy. In addition, CBP is a tumour-promoting molecule for some leukaemia types and is broadly studied in AML without *MLLr*, where aberrantly expressed CBP acetylates oncogenic transcription factors (such as MYB and CREB) to increase their transcriptional activity, contributing to leukemogenesis [35]. The fusion of the *CBP* gene with the *MLL* gene is well known to lead to MLL-CBP fusion, which drives the therapy-related leukaemia [36, 37]. Furthermore, it has been reported that MLL and CBP, together with CREB or MYB, form a ternary complex in vitro, and inhibitors targeting the MYB:CBP/P300 complex are very efficient at suppressing the survival of leukaemia cells harbouring *MLLr* [38, 39]. Taken together, these studies suggest that, in *MLLr* leukaemia without CBP translocation, CBP may participate in the super complex containing MLL-fusion and DOT1L, where CBP directly acetylates DOT1L to promote its stability. This view is supported by our findings that CBP deficiency blocks cell growth and increases proteasomal degradation of the DOT1L protein in *MLLr* leukaemia.

While studies have focused on the potent acetyltransferase activity and inhibition of acetyltransferase in treating various cancers, the regulation of CBP expression and homoeostasis have rarely been studied. Previous studies have indicated that proteasomes can degrade CBP in HeLa cells, 3T3 fibroblasts, lung epithelial cells and neural cells [40–44]. In the present study, CBP is rapidly degraded by the proteasome in *MLLr* leukaemia, and S5178 phosphorylation by ATM confers CBP stability. While studies have shown that phosphorylation at S1382/S1386 by I-Kappa-B kinase alpha (IKKA) increases the acetyltransferase and transcriptional activities of CBP to promote tumour cell growth [45], no study has reported the stability-associated posttranslational protein modification of CBP, except for ubiquitination. Although without exploring of the reason why S5178 phosphorylation prevents CBP degradation, we speculated that the phosphorylated CBP binds less to E3 ubiquitin ligases or more to deubiquitinating enzymes. Several E3 ubiquitin ligases and deubiquitinating enzymes, such as MDM2, FBXL19 and USP14, have been found for CBP [41, 45]. All these findings need to be further explored in *MLLr* leukaemia.

### The role of ATM beyond the artificially induced DNA damage response

ATM has long been considered the kinase responsible for initiating the DNA damage response (DDR) following DNA double-strand breaks (DSBs), and the DNA damage-activated phosphorylation of ATM(S1981) has been proven to be the essential element for the phosphorylation of downstream targets in the DDR [26, 27, 46, 47]. As such, few studies concerning ATM have been performed without describing the DDR. Typically, for studies with in vivo animal models or in vitro cell cultures, DSBs are artificially introduced by ion radiation or DNA-damaging reagents used for cancer treatment. Although DNA lesions occur ubiquitously within a cell, artificially introduced DSBs are excellent models for investigating ATM activation and DDR; however, they may conceal some fundamental roles of the ATM protein, as no such massive DSBs are generated under normal physiological conditions [48]. Without artificial DSB introduction, we characterised the kinase activity of ATM for the phosphorylation of CBP(S1578), which can be activated by DSBs generated endogenously by reactive oxygen species (ROS) or stalled replication forks. However, when artificial DSBs were introduced, the upregulated activation of ATM kinase did not turn into the phosphorylation of CBP. In addition, the kinase-dead mutant of ATM, ATM(S1981A), increased the phosphorylation of CBP both in vivo and in vitro. Our data revealed that DSB activation- and S1981-independent kinase activity are responsible for CBP phosphorylation in ATM, which is consistent with and serves as a demonstration of the view that ATM kinase regulates the cellular response to more than genotoxic stress [49, 50].

DDR involves numerous kinases that are potential cancer therapy targets [51, 52]. As one of the hearts of the DDR and the master regulator of the DSB response in the cell, ATM kinase has long been considered a promising target for cancer treatment [9]. As the initial ATM inhibitor and lead structure for pharmacological development, KU-55933 potently inhibits ATM kinase [53, 54]. We confirmed the kinase inhibition of ATM by KU-55933 and found that KU-55933 blocked the phosphorylation of CBP and the subsequent regulation of DOT1L by ATM in 293T cells. Furthermore, in human *MLLr*-AML cells, treatment with KU-55933 downregulated the expression of the CBP-DOT1L axis and led to retarded cell growth (data not shown). These results demonstrated the essential kinase activity of ATM for CBP-DOT1L regulation.

Our study indicated that ATM is essential for the long-term maintenance of leukaemia stem cells (LSCs) in *MLLr*-AML. LSCs can self-renew and are thought to be one of the critical reasons for the relapse of refractory leukaemia, including *MLLr*-AML [55–57]. A previous study demonstrated that the loss of ATM did not impact the development of murine AML driven by MLL-AF9 in primary transplants; however, the inhibition of ATM significantly alleviated the disease burden and extended the survival of leukaemia mice [12]. A recent study revealed that combining DNA-damaging agents with valosin-containing protein inhibitors critical for ATM kinase activation impaired leukaemia growth in a murine AML model driven by MLL-AF9 [58]. In our study, the induction of murine *MLLr*-AML with ATM loss was also successful in primary transplants (F0 recipients); however, the subsequent LSC transplantation assays (F1 and F2 recipients) demonstrated the essential role of ATM in maintaining the self-renewal ability of LSCs in *MLLr*-AML.

After the initial identification of ATM inhibitor (KU-55933) two decades ago, considerable effort has been devoted to developing the next generations for clinical use [54]. Notably, the newest class of selective ATM inhibitors (M4076) has been tested in clinical trials to treat advanced solid tumours (NCT04882917). In *MLLr*-AML, although several impactful preclinical studies have demonstrated that the inhibition of ATM was a potent therapeutics, ATM inhibitors have not been tested at the clinical level [11, 12, 58]. Given the remarkable therapeutic effects in treating cancer, it is somewhat unexpected that ATM inhibitors have not been widely used in clinical. Possibly, most current studies concerning the combination of DNA-damaging agents depended so heavily on the DNA damage response exhibited by ATM kinase, in which the DNA damage-independent roles of ATM were embedded. As both CBP and DOT1L inhibitors are under development for clinical use [59, 60], our study indicates that inhibiting ATM alone or combined with inhibitors targeting CBP or DOT1L would be an advanced approach for treating *MLLr*-AML.

In summary, we propose that ATM kinase plays dual roles in *MLLr-AML* (Supplementary Fig. S7J). On the one hand, the phosphorylation of ATM(S1981) is required for the DNA damage response and the repair of DSBs in *MLLr-AML* cells. On the other hand, a DSB-activation or ATM(S1981)-independent pathway exists. ATM phosphorylates CBP(S1578) to maintain its stability; the stabilised CBP then functions in DOT1L acetylation and subsequent H3K79 methylation to maintain leukaemia stem cells and leukaemia cells in *MLLr-AML*. Loss of the *ATM* gene or kinase inhibition of ATM promotes proteasomal degradation of the CBP protein and breakage of DOT1L-H3K79me signalling, ultimately leading to exhaustion of *MLLr-AML* cells. Our study revealed the role of the ATM-CBP-DOT1L axis in *MLLr-AML* and broadened the view of DDR enzymes as potent targets for cancer therapy.

## Materials and methods

### Animals

C57BL/6 mice and *ATM*^+/−^ mice [61] were maintained at the specific pathogen-free animal facility of Tongji University. All animal procedures were performed according to protocols approved by the Animal Committee of the School (Tongji University, Shanghai, China, TJmed-010-10). The *ATM*^+/−^ mice were crossed to obtain *ATM*^+/+^ and *ATM*^−/−^ mice, and genotyping was performed according to a standard protocol provided by The Jackson Laboratory (Protocol 36208). For animal models, the male C57BL/6 mice at the age of 6–8 weeks, and the male or female *ATM*^*+/+*^ and *ATM*^*−/−*^ mice at the age of 6–8 weeks were used.

### Cells

All cell lines used in this study were obtained from the American Type Culture Collection (ATCC). 293T cells were kept in Dulbecco’s modified Eagle’s medium (DMEM) supplemented with 10% (vol/vol) FBS (fetal bovine serum). MOLM-13 cells were grown in Roswell Park Memorial Institute (RPMI) 1640 medium supplemented with 10% (vol/vol) FBS. MV4-11 cells were maintained in ISCove’s modified Dulbecco’s medium (IMDM) medium supplemented with 10% (vol/vol) FBS. Murine cells isolated from mouse bone marrow and sorted by flow cytometry were grown in RPMI 1640 medium supplemented with 10% (vol/vol) FBS, IL-3 (interleukin-3, 10 ng/mL), IL-6 (interleukin-6, 10 ng/mL), SCF (stem cell factor, 10 ng/mL) and G-CSF (granulocyte colony-stimulating factor, 10 ng/mL). Penicillin (100 IU/mL) and streptomycin (100 μg/mL) were added to the medium during all culture procedures. All cells were maintained in a humidified incubator equilibrated with 5% CO2 at 37 °C. All cells were tested free of mycoplasma.

### Plasmids

pCMV-dR8.2 (Addgene plasmid #8455) and pCMV-VSV-G (Addgene plasmid #8454) were gifts from Bob Weinberg [62]. pCL-Eco (Addgene plasmid #12371) was a gift from Inder Verma [63]. The original pMIG-FLAG-MLL-AF9 (Addgene plasmid #71443) was a gift from Daisuke Nakada [64], and the EGFP was replaced with YFP. The original pLVX-shRNA2-ZsGreen1 (No. 632179) was purchased from Clontech, and ZsGreen1 was replaced with puromycin N-acetyltransferase to obtain pLVX-shRNA2-Puro. The scrambled (shSCR) and *ATM* or *CBP-*targeted (shATM or shCBP) shRNAs were inserted into pLVX-shRNA2-ZsGreen1 or pLVX-shRNA2-Puro. pcDNA3.1(+)-Flag-His-ATM (Addgene plasmid #31985) and hATMS1981A (Addgene plasmid # 32300) were gifts from Michael Kastan [26, 27]. The original MSCB-hDot1Lwt (Addgene plasmid #74173) was a gift from Yi Zhang [65]. *DOT1L* from MSCB-hDot1Lwt was subcloned and inserted into pCDNA3.1-3×HA-N (P0160, miaolingbio, Wuhan, China) and pCDNA3.1-FLAG-N (P0794, miaolingbio, Wuhan, China) to generate pCDNA3.1-3×HA-DOT1L and pCDNA3.1-FLAG-DOT1L, respectively. HA-DOT1L(K358Q) and HA-DOT1L(K358R) were generated from pCDNA3.1-3×HA-DOT1L using a Fast Site-Directed Mutagenesis Kit (KM101, TIANGEN Biotech, Beijing, China). pEnCMV-CBP(human)-3×FLAG was purchased from miaolingbio (P32329). *CBP* from pEnCMV-CBP(human)-3×FLAG was subcloned and inserted into pCDNA3.1-3×HA-C (P6808, miaolingbio, Wuhan, China) to generate pCDNA3.1-CBP-3×HA. All CBP mutants were generated from pCDNA3.1-3×HA-DOT1L using a Fast Site-Directed Mutagenesis Kit (KM101, TIANGEN Biotech, Beijing, China). pCDNA3.1-3×HA-UB (P10697) was purchased from Miaolingbio, and 3×HA was replaced with Myc to obtain pCDNA3.1-Myc-UB. pCDNA3.1-Myc-UB-K6/K11/K27/K29/K33/K48/K63 were generated from pCDNA3.1-Myc-UB by mutating all the lysine residues except for the indicated one in the ubiquitin chain with the Fast Site-Directed Mutagenesis Kit. The original pLVX-IRES-ZsGreen1 (No. 632187) was purchased from Clontech, and ZsGreen1 was replaced with puromycin N-acetyltransferase to obtain pLVX-IRES-Puro. HA-DOT1L and HA-CBPΔ were amplified from pCDNA3.1-3×HA-DOT1L and pCDNA3.1-CBP-3×HA, respectively, and inserted into pLVX-IRES-Puro to generate pLVX-HA-DOT1L-IRES-Puro and pLVX-HA-CBPΔ-IRES-Puro, respectively.

### Antibodies and chemical reagents

APC Mouse Lineage Antibody Cocktail (BD Biosciences #558074), Sca-1 as PE-Cy™7 Rat Anti-Mouse Ly-6A/E (BD Biosciences #558162), c-KIT as PE Rat Anti-Mouse CD117 (BD Biosciences #553355), Mouse Anti-ATM (Abcam, #ab78, WB 1:1000), Rabbit Anti-ATM (phospho S1981) (Abcam, #ab81292, WB 1:1000),

Rabbit Anti-DOT1L (abclonal, #A12329, WB 1:1000, IP 1:100), Rabbit Anti-H3K79me2 (Abcam, #ab3594, WB 1:1000), Rabbit Anti-Ubiquitin (Santa Cruz, #sc-8017, WB 1:1000), Rabbit Anti-ACTB (Cell Signalling Technology, #4970, WB 1:1000), Mouse Anti-ACTB (ZENBIO, #250174, WB 1:1000), Rabbit Anti-DOT1L(K358Ace) (PTM BIO, a gift from Weiguo Zhu lab, Shenzhen University, WB, 1:1000), Mouse Anti-O-GlcNAcylation (Santa Cruz, #sc-59623, WB 1:1000),

Rabbit Anti-CBP (Cell Signalling Technology, #7389, WB 1:1000, IP 1:100), Mouse Anti-CBP (Santa Cruz, #sc-365387 AC, IP:50), Rabbit Anti-K48-linkage Specific Polyubiquitin (Cell Signalling Technology, #8081, WB 1:1000), Rabbit Anti-phospho-S/TQG motif (Cell Signalling Technology, #6966, WB 1:1000), Rabbit Anti-γH2AX (Abcam, #ab81299, WB 1:1000), Mouse Anti-HA (Santa Cruz, #sc-7392, WB 1:1000, IP:100), Rabbit Anti-HA (Santa Cruz, #sc-805, WB 1:1000, IP:100), Rabbit Anti-Myc (Cell Signalling Technology, #2276, WB 1:1000, IP 1:100), Rabbit Anti-Flag (Cell Signalling Technology, #14793, WB 1:1000, IP 1:100), Mouse Anti-Flag (Cell Signalling Technology, #8146, WB 1:1000, IP 1:100), Mouse Anti-His (Sangon, D191001, WB 1:1000), Mouse normal IgG (Santa Cruz, #sc-2025, IP:100), Rabbit normal IgG (Santa Cruz, #sc-2027, IP:100), HRP-linked anti-Rabbit IgG (Cell Signalling Technology, #7074, WB 1:2000), HRP-linked anti-Mouse IgG (Cell Signalling Technology, #7076, WB 1:2000), Protein A/G PLUS-Agarose (Santa Cruz, #sc-2003, IP:50), ECL reagents (Epizyme Biotech, #SQ101, SQ201, SQ202), Cycloheximide (CHX, MedChemExpress, #HY-12320), MG132 (Selleck, #2619), Chloroquine (CHQ, Selleck, #6999), KU-55933 (Abcam, #ab120637), Etoposide (ETO, MedChemExpress, # HY-13629), Hydroxyurea (HU, Sigma-Aldrich, #H8627).

### Synthesised DNA oligos

All DNA sequences were synthesised by Sangon Biotech (Shanghai, China). The sequences of the shRNAs and primers used for quantitative PCR were as follows:

shSCR, 5′-TTCTCCGAACGTGTCACGT-3′;

shCBP-1, 5′-GGCCTCCTCAATAGTAACT-3′;

shCBP-2, 5′-GGAAGGGTGGATTGATGTTTA-3′;

shATM-1, 5′-CCTTTCATTCAGCCTTTAGAA-3′;

shATM-2, 5′-TGAAGATGGTGCTCATAAA-3′;

mouse *Gapdh* forward 5′-AGGTCGGTGTGAACGGATTTG-3′,

reverse 5′-TGTAGACCATGTAGTTGAGGTCA-3′;

mouse *Cd34* forward 5′-AAGGCTGGGTGAAGACCCTTA-3′

reverse 5′-TGAATGGCCGTTTCTGGAAGT-3′;

mouse *Cd38* forward 5′-TCTCTAGGAAAGCCCAGATCG-3′

reverse 5′-GTCCACACCAGGAGTGAGC-3′;

mouse *Hoxa9* forward 5′-CCCCGACTTCAGTCCTTGC-3′

reverse 5′-GATGCACGTAGGGGTGGTG-3′;

mouse *Hoxa10* forward 5′-CCTGCCGCGAACTCCTTTT-3′

reverse 5′-GGCGCTTCATTACGCTTGC-3′;

mouse *Pbx3* forward 5′-CGAGGCGCAAGCAAAGAAAC-3′

reverse 5′-TGCCAAAAGCATATTGTCCAGT-3′;

mouse *Meis1* forward 5′-GCAAAGTATGCCAGGGGAGTA-3′

reverse 5′-TCCTGTGTTAAGAACCGAGGG-3′;

mouse *Dot1l* forward 5′-GAGGCTCAAGTCGCCTGTG-3′

reverse 5′-GACCCACCGGATAGTCTCAAT-3′;

mouse *Cbp* forward 5′-GGCTTCTCCGCGAATGACAA-3′

reverse 5′-GTTTGGACGCAGCATCTGGA-3′;

human *ATM* forward 5′-TTGATCTTGTGCCTTGGCTAC-3′

reverse 5′-TATGGTGTACGTTCCCCATGT-3′;

human *DOT1L* forward 5′-AATCCCGGATCTCAAGCTCG-3′

reverse 5′-GCACGGTTGTACTTGTCGC-3′;

human *CBP* forward 5′-CAACCCCAAAAGAGCCAAACT-3′

reverse 5′-CCTCGTAGAAGCTCCGACAGT-3′;

human *GAPDH* forward 5′-GGAGCGAGATCCCTCCAAAAT-3′

reverse 5′-GGCTGTTGTCATACTTCTCATGG-3′.

### Virus production and transduction of target cells

The viruses were produced in 293T cells according to a standard protocol. Briefly, 10 μg of expressing plasmid with 7.5 μg of pCMV-dR8.2 and 5 μg of pCMV-VSV-G lentiviral packaging plasmid were cotransfected into 293T cells in a 100 mm tissue dish with 45 μl of Lipo293^TM^ (Beyotime Biotechnology C0521, Shanghai, China) transfection reagent for lentivirus production. Thirty micrograms of expressing plasmid with 15 μg of PCL-ECO retroviral packaging plasmid were co-transfected into 293T cells in a 100 mm tissue dish with 90 μl of Lipo293^TM^ transfection reagent for retrovirus production. Then, 48 h and 72 h posttransfection, the medium of the transfected 293T cells containing the retrovirus or lentivirus was collected and filtered through a 0.45 μm filter. NaCl was added to the medium to a final concentration of 0.3 M, and polyethene glycerol (PEG) 6000 was added to a final concentration of 8.5% (vol/vol). The mixed virus medium was incubated at 4 °C for 2 h and mixed well every 30 min. The virus medium was centrifuged at 7500 × *g* for 2 h at 4 °C. The supernatant was discarded, and the pellets were suspended in the appropriate medium. The concentrated virus was titrated using quantitative PCR. The target cells were suspended in culture medium containing the proper virus and polybrene (10 ng/mL) for virus transduction. The cell suspensions were plated into 6/12-well plates and subjected to horizontal centrifugation at 400 × *g* for 2 h at room temperature before they were returned to the incubator.

### Induction and analysis of murine AML

The generation of murine AMLs has been reported previously [66]. For F0 recipient induction, the mice were sacrificed by cervical dislocation. Then, the femurs were removed and washed with ice-cold phosphate-buffered saline (PBS) containing 1% penicillin/streptomycin (PS) in a tissue culture dish. Cells from the bone marrow were collected in ice-cold PBS containing 1% (PS) and stained with antibodies (Linage, Sca-1 and c-KIT) following the manufacturer’s instructions. The LSKs (Lin^-^/Sca-1^+^/c-KIT^+^) were collected by flow cytometry, cultured for three days, and ectopically expressed YFP/MLL-AF9 via retrovirus transduction. The transduced cells were cultured for three days, after which the YFP^+^ cells were collected. A total of 3,000 YFP^+^ cells with 500,000 bone marrow cells from wild-type C57BL/6 mice were injected into the caudal vein of lethally irradiated (10 Gy) wild-type C57BL/6 mice. For the subsequent F1 generation, LSCs (YFP^+^/c-kit^+^) were isolated from F0 recipient mice. Then, 1,000/500/100 LSCs in combination with 500,000 bone marrow cells from wild-type C57BL/6 mice were injected into the caudal vein of lethally irradiated wild-type C57BL/6 mice. Isolated LSCs from F1 recipients were used to generate F2 recipients.

For peripheral blood (PB) analysis, approximately 10 μl of PB from the tail vein of each recipient mouse was collected in a 1.5 ml tube containing 2 mM EDTA as an anticoagulant. The PB samples were washed twice with PBS, and the cells were collected by centrifugation at 2000 × *g* for 3 min. Cells from wild-type C57BL/6 mice were obtained under the same conditions and were used as the negative control. All the samples were subjected to flow cytometry for YFP% analysis within 1 h.

For histopathology analysis, the mice were sacrificed by cervical dislocation, and the spleens and livers were collected. The samples were then fixed in formalin and embedded in paraffin/formalin blocks. The tissue sections were collected, and H&E staining was performed using an H&E Staining Kit (Beyotime Biotechnology C0105, Shanghai, China) according to the manufacturer’s protocol.

### Comet and cell viability assays

The LSCs isolated from F2 recipients were cultured in RPMI 1640 medium supplied supplemented with 10% (vol/vol) FBS, IL-3 (interleukin-3, 10 ng/mL), IL-6 (interleukin-6, 10 ng/mL), SCF (stem cell factor, 10 ng/mL) and G-CSF (granulocyte colony-stimulating factor, 10 ng/mL). For irradiation, the cells in tissue culture plates were placed within the X-ray generating equipment (RS2000pro, Rad Source Technologies, USA). The comet assay was performed with CometAssay® Kits (Trevigen, Catalog # 4250-050-K) following a standard protocol. Quantification was performed using a Comet Assay IV (Instem, UK). An inhibitor-mediated cell viability assay was performed with EPZ004777 (MedChemExpress, #HY-15227) following published methods [7]. The number of viable cells was detected with an Enhanced Cell Counting Kit-8 (Beyotime Biotechnology C0042, Shanghai, China). The IC50 values were calculated from the concentration-dependence curves with GraphPad Prism.

### RNA extraction and quantitative PCR

Total RNA was extracted using TRIzol reagent (Invitrogen, #15596026) following standard procedures. Reverse transcription was performed using the TransScript All-in-One Kit (TransGen #AT341, Beijing, China). Quantitative PCR was performed with SuperMix (TransGen #AQ621, Beijing, China) in an ABI Prism 7500 device. The results were analysed using the 2^-ΔΔCT^ method [67].

### RNA-Seq and online data analysis

RNA-Seq and data analysis were performed following standard procedures. Total RNA was extracted using TRIzol reagent (Invitrogen #15596026). The RNA was qualified and quantified using a NanoDrop and Agilent 2100 bioanalyser. The mRNA was then purified using oligo (dT)-attached beads. cDNA libraries were obtained after mRNA fragmentation, cDNA synthesis and PCR amplification. cDNA libraries were analysed with an Agilent 2100 bioanalyser and sequencing was performed using an Illumina Hiseq 2000. The raw sequence data were qualified and quantified by FastQC (https://www.bioinformatics.babraham.ac.uk/projects/fastqc) to obtain clean reads. The clean reads in FASTQ format were deposited in the Sequence Read Archive (PRJNA954265, SRR24126271, SRR24126270, SRR24126269, SRR24126268). The clean reads were aligned to reference genomes using HISAT2 (http://www.ccb.jhu.edu/software/hisat/index.shtml) [68] and aligned to the reference coding gene set using Bowtie2 (https://bowtie-bio.sourceforge.net/bowtie2/index.shtml) [69]. The gene expression was calculated by RSEM (https://github.com/deweylab/RSEM) [70]. Differential gene expression analysis was performed using DESeq2 (http://www.bioconductor.org/packages/release/bioc/html/DESeq2.html) [71]. The differentially expressed genes were then subjected to Kyoto Encyclopedia of Genes and Genomes (KEGG) enrichment analysis using DAVID (https://david.ncifcrf.gov/) [72, 73]. The Gene set enrichment analysis (GSEA) was subsequently performed (https://www.gsea-msigdb.org/gsea/index.jsp) [18, 19].

The “BloodPool: AML samples with normal cells” dataset from BloodSpot (https://servers.binf.ku.dk/bloodspot/) was used for gene expression analysis of various AML patients [74]. The dataset included human AML cells from the GSE13159, GSE15434, GSE61804, and GSE14468 cohorts and The Cancer Genome Atlas (TCGA). The LAML dataset from the TCGA cohort was selected for overall survival analysis with custom group cut-offs of 60% high and 40% low in the Gene Expression Profiling Interactive Analysis (GEPIA)2 (Version 2) (http://gepia2.cancer-pku.cn/#survival) [75].

### Immunoprecipitation (IP)

The cells were harvested, washed with ice-cold PBS and lysed in cell lysis buffer (25 mM Tris-HCl pH 7.4, 150 mM NaCl, 1 mM EDTA, 1% NP-40, 1% sodium deoxycholate, and 0.1% SDS) supplemented with protease inhibitor (PMSF, 1 mM, Beyotime Biotechnology ST506, Shanghai, China) and protein phosphatase inhibitor (Beyotime Biotechnology P1082, Shanghai, China) for phosphorylation detection, deacetylase inhibitor (Beyotime Biotechnology P1112, Shanghai, China) for acetylation detection and deubiquitinase inhibitor (PR-619, 5 μM, MedChemExpress HY-13814, Shanghai, China) for ubiquitin detection. The cell lysates were sonicated and centrifuged at 14,000 × *g* for 15 min at 4 °C. The cleared lysates were then incubated with primary antibody overnight at 4 °C with shaking, followed by incubation with protein A/G agarose beads (Santa Cruz Biotechnology, sc-2003) at 4 °C with shaking for 4-8 h. Next, the precipitated beads were washed with IP washing buffer (25 mM Tris-HCl pH 7.4, 150 mM NaCl, 1 mM EDTA, 1% NP-40, 5% glycerol) four times. Then, the precipitated proteins were eluted with SDS sample buffer and boiled for 5–10 min, followed by SDS-PAGE and western blotting.

### Coimmunoprecipitation (Co-IP)

The cells were harvested, washed with ice-cold PBS, and lysed in cell lysis buffer for Western blotting and IP (Beyotime Biotechnology P0013, Shanghai, China). The cell lysates were centrifuged at 14,000 × *g* for 15 min at 4 °C. The cleared lysates were then incubated with primary antibody or control IgGs overnight at 4 °C with shaking, followed by protein A/G agarose beads (Santa Cruz Biotechnology, sc-2003) at 4 °C with shaking for 4–8 h. The precipitated beads were washed with IP washing buffer (25 mM Tris-HCl pH 7.6, 150 mM NaCl, 1 mM EDTA, 1% NP-40, 5% glycerol) four times. Then, the precipitated proteins were eluted with SDS sample buffer and boiled for 5–10 min, followed by SDS-PAGE and western blotting.

### Tag protein purification from 293T cells

293T cells transfected with plasmids expressing HA-CBP or Flag-ATM were grown for 3 days, harvested, washed with ice-cold PBS and lysed in cell lysis buffer for Western blotting and IP (Beyotime Biotechnology P0013, Shanghai, China). The cell lysates were sonicated and centrifuged at 14,000 × *g* for 15 min at 4 °C. The cleared lysates were then incubated with HA or Flag antibodies overnight at 4 °C with shaking, followed by protein A/G agarose beads (Santa Cruz Biotechnology, sc-2003) at 4 °C with shaking for 4–8 h. The precipitated beads were washed with IP washing buffer (25 mM Tris-HCl pH 7.6, 150 mM NaCl, 1 mM EDTA, 1% NP-40, 5% glycerol) four times. Then, the precipitated proteins were eluted with HA (Beyotime Biotechnology, P9808, Shanghai, China) or Flag (Beyotime Biotechnology P9801, Shanghai, China) peptide (150 μg/ml) in TBS buffer (50 mM Tris-HCl pH 7.4, 150 mM NaCl).

### In vitro dephosphorylation and phosphorylation assay

For dephosphorylation, HA-CBP was purified from 293T cells using the same procedure before the precipitated beads were eluted, as described above for tag protein purification. The precipitated beads were washed three times with TBS buffer (50 mM Tris-HCl pH 7.4, 150 mM NaCl) after washing with IP washing buffer. The beads were then suspended in TBS buffer and subjected to dephosphorylation with Lambda Protein Phosphatase (New England Biolabs, P0753) for 1–3 h at 30 °C. The beads were washed three times with IP washing buffer (25 mM Tris-HCl pH 7.6, 150 mM NaCl, 1 mM EDTA, 1% NP-40, 5% glycerol) and suspended in TBS buffer.

For phosphorylation, the eluted or bead-bound HA-CBP was incubated with or without purified Flag-ATM in kinase assay buffer (25 mM Tris-HCl pH 7.4, 2 mM DTT, 10 mM MgCl2, 10 mM MnCl2, 1 mM ATP) for 1 h at 30 °C. The samples were boiled in SDS sample buffer and analysed by SDS-PAGE and western blotting.

### Western blotting

The cells were harvested, washed with ice-cold PBS and lysed in cell lysis buffer (25 mM Tris-HCl pH 7.6, 150 mM NaCl, 1 mM EDTA, 1% NP-40, 1% sodium deoxycholate, 0.1% SDS) supplemented with protease inhibitor (PMSF, 1 mM, Beyotime Biotechnology ST506, Shanghai, China) and protein phosphatase inhibitor (Beyotime Biotechnology P1082, Shanghai, China) for phosphorylation detection. The cell lysates were sonicated and centrifuged at 14,000 × *g* for 15 min at 4 °C. The cleared lysates were then boiled in SDS sample buffer for 5–10 min. The boiled total lysates were size fractionated by SDS-PAGE with 8% or 4–12% gels (Sangon, SimplePAGE C691103 and C691100, Shanghai, China). The blots were incubated first with specific antibodies and then with horseradish peroxidase-conjugated secondary antibodies, followed by visualisation with an enhanced chemiluminescence (ECL) detection system (Clinx ChemiScope 6100, Shanghai, China). The boiled samples from the IP, Co-IP, and in vitro phosphorylation assays were subjected to the same procedure.

### Data collection, quantification and statistical analyses

The investigators were blinded to the group allocations during the data collection and no randomisation was used for animal studies. No blinding was done for in vitro studies with cellular and molecular assays. The sample size was chosen to ensure adequate power for statistical analyses following the standard published studies. The stem cell frequency was analysed using the online tool ELDA (https://bioinf.wehi.edu.au/software/elda/index.html) [16]. The flow cytometry results were analysed by CytExpert and FlowJo. The quantification of the band intensity in the western blotting results was performed with ImageJ. A pre-established criteria was used to exclude the data beyond the normal distribution, and no data was excluded during the analysis in this study. The exact sample size (n) of each group was manifested in the specific part of the figure. The statistical analysis was performed with GraphPad. Unless otherwise indicated, all the data are represented as the means ± SEMs of at least three independent experiments. The statistical significance of the differences was determined by the inferred analyses in the figure legends, and *p* < 0.05 was considered to indicate statistical significance.

### Supplemental information

The supplemental information includes supplemental figures and legends.

### Supplementary information

Supplementary figures and legends

## Data Availability

The RNA-seq data are available in the Sequence Read Archive (PRJNA954265, SRR24126271, SRR24126270, SRR24126269, SRR24126268). The data and materials relevant to this study are available from the corresponding authors upon reasonable request.

## References

[1] Vardiman JW, Thiele J, Arber DA, Brunning RD, Borowitz MJ, Porwit A (2009). The 2008 revision of the World Health Organization (WHO) classification of myeloid neoplasms and acute leukemia: rationale and important changes. Blood.

[2] Meyer C, Burmeister T, Groger D, Tsaur G, Fechina L, Renneville A (2018). The MLL recombinome of acute leukemias in 2017. Leukemia.

[3] Chen CW, Armstrong SA (2015). Targeting DOT1L and HOX gene expression in MLL-rearranged leukemia and beyond. Exp Hematol.

[4] Slany RK (2016). The molecular mechanics of mixed lineage leukemia. Oncogene.

[5] Bernt KM, Zhu N, Sinha AU, Vempati S, Faber J, Krivtsov AV (2011). MLL-Rearranged Leukemia Is Dependent on Aberrant H3K79 Methylation by DOT1L. Cancer Cell.

[6] Daigle SR, Olhava EJ, Therkelsen CA, Basavapathruni A, Jin L, Boriack-Sjodin PA (2013). Potent inhibition of DOT1L as treatment of MLL-fusion leukemia. Blood.

[7] Daigle SR, Olhava EJ, Therkelsen CA, Majer CR, Sneeringer CJ, Song J (2011). Selective killing of mixed lineage leukemia cells by a potent small-molecule DOT1L inhibitor. Cancer Cell.

[8] Perner F, Gadrey JY, Xiong YJ, Hatton C, Eschle BK, Weiss A (2020). Novel inhibitors of the histone methyltransferase DOT1L show potent antileukemic activity in patient-derived xenografts. Blood.

[9] Blackford AN, Jackson SP (2017). ATM, ATR, and DNA-PK: The Trinity at the Heart of the DNA Damage Response. Mol Cell.

[10] Blanpain C, Mohrin M, Sotiropoulou PA, Passegue E (2011). DNA-Damage Response in Tissue-Specific and Cancer Stem Cells. Cell Stem Cell.

[11] Santos MA, Faryabi RB, Ergen AV, Day AM, Malhowski A, Canela A (2014). DNA-damage-induced differentiation of leukaemic cells as an anti-cancer barrier. Nature.

[12] Morgado-Palacin I, Day A, Murga M, Lafarga V, Anton ME, Tubbs A, et al. Targeting the kinase activities of ATR and ATM exhibits antitumoral activity in mouse models of MLL-rearranged AML. Sci Signal. 2016;9:ra91. 10.1126/scisignal.aad8243 PMC5066844 27625305

[13] Wood K, Tellier M, Murphy S. DOT1L and H3K79 Methylation in Transcription and Genomic Stability. Biomolecules. 2018;8:11. 10.3390/biom8010011 PMC5871980 29495487

[14] Ljungman M, Parks L, Hulbatte R, Bedi K (2019). The role of H3K79 methylation in transcription and the DNA damage response. Mutat Res Rev Mutat.

[15] Skucha A, Ebner J, Schmöllerl J, Roth M, Eder T, César-Razquin A (2018). MLL-fusion-driven leukemia requires SETD2 to safeguard genomic integrity. Nat Commun.

[16] Hu YF, Smyth GK (2009). ELDA: Extreme limiting dilution analysis for comparing depleted and enriched populations in stem cell and other assays. J Immunol Methods.

[17] Hess JL, Bittner CB, Zeisig DT, Bach C, Fuchs U, Borkhardt A (2006). c-Myb is an essential downstream target for homeobox-mediated transformation of hematopoietic cells. Blood.

[18] Subramanian A, Tamayo P, Mootha VK, Mukherjee S, Ebert BL, Gillette MA (2005). Gene set enrichment analysis: a knowledge-based approach for interpreting genome-wide expression profiles. Proc Natl Acad Sci USA.

[19] Mootha VK, Lindgren CM, Eriksson KF, Subramanian A, Sihag S, Lehar J (2003). PGC-1alpha-responsive genes involved in oxidative phosphorylation are coordinately downregulated in human diabetes. Nat Genet.

[20] Brown AL, Wilkinson CR, Waterman SR, Kok CH, Salerno DG, Diakiw SM (2006). Genetic regulators of myelopoiesis and leukemic signaling identified by gene profiling and linear modeling. J Leukoc Biol.

[21] Mu JJ, Wang Y, Luo H, Leng M, Zhang J, Yang T (2007). A proteomic analysis of ataxia telangiectasia-mutated (ATM)/ATM-Rad3-related (ATR) substrates identifies the ubiquitin-proteasome system as a regulator for DNA damage checkpoints. J Biol Chem.

[22] Bensimon A, Schmidt A, Ziv Y, Elkon R, Wang SY, Chen DJ (2010). ATM-dependent and -independent dynamics of the nuclear phosphoproteome after DNA damage. Sci Signal.

[23] Liu CH, Yang QY, Zhu Q, Lu XP, Li MT, Hou TY (2020). CBP mediated DOT1L acetylation confers DOT1L stability and promotes cancer metastasis. Theranostics.

[24] Song TJ, Zou QL, Yan YY, Lv SL, Li N, Zhao XF, et al. DOT1L O-GlcNAcylation promotes its protein stability and MLL-fusion leukemia cell proliferation. Cell Rep. 2021;36:109739. 10.1016/j.celrep.2021.109739 34551297

[25] Thrower JS, Hoffman L, Rechsteiner M, Pickart CM (2000). Recognition of the polyubiquitin proteolytic signal. EMBO J.

[26] Canman CE, Lim DS, Cimprich KA, Taya Y, Tamai K, Sakaguchi K (1998). Activation of the ATM kinase by ionizing radiation and phosphorylation of p53. Science.

[27] Bakkenist CJ, Kastan MB (2003). DNA damage activates ATM through intermolecular autophosphorylation and dimer dissociation. Nature.

[28] Tsai CT, So CWE (2017). Epigenetic therapies by targeting aberrant histone methylome in AML: molecular mechanisms, current preclinical and clinical development. Oncogene.

[29] Lacoste N, Utley RT, Hunter JM, Poirier GG, Cote J (2002). Disruptor of telomeric silencing-1 is a chromatin-specific histone H3 methyltransferase. J Biol Chem.

[30] Nguyen AT, Taranova O, He J, Zhang Y (2011). DOT1L, the H3K79 methyltransferase, is required for MLL-AF9-mediated leukemogenesis. Blood.

[31] Chang MJ, Wu HY, Achille NJ, Reisenauer MR, Chou CW, Zeleznik-Le NJ (2010). Histone H3 Lysine 79 Methyltransferase Dot1 Is Required for Immortalization by MLL Oncogenes. Cancer Res.

[32] Deshpande AJ, Chen LY, Fazio M, Sinha AU, Bernt KM, Banka D (2013). Leukemic transformation by the MLL-AF6 fusion oncogene requires the H3K79 methyltransferase Dot1l. Blood.

[33] Stein EM, Garcia-Manero G, Rizzieri DA, Tibes R, Berdeja JG, Savona MR (2018). The DOT1L inhibitor pinometostat reduces H3K79 methylation and has modest clinical activity in adult acute leukemia. Blood.

[34] Stauffer F, Weiss A, Scheufler C, Mobitz H, Ragot C, Beyer KS (2019). New Potent DOT1L Inhibitors for in Vivo Evaluation in Mouse. ACS Med Chem Lett.

[35] Dutta R, Tiu B, Sakamoto KM (2016). CBP/p300 acetyltransferase activity in hematologic malignancies. Mol Genet Metab.

[36] Rowley JD, Reshmi S, Sobulo O, Musvee T, Anastasi J, Raimondi S (1997). All patients with the T(11;16)(q23;p13.3) that involves MLL and CBP have treatment-related hematologic disorders. Blood..

[37] Sobulo OM, Borrow J, Tomek R, Reshmi S, Harden A, Schlegelberger B (1997). MLL is fused to CBP, a histone acetyltransferase, in therapy-related acute myeloid leukemia with a t(11;16)(q23;p13.3). Proc Natl Acad Sci USA.

[38] Goto NK, Zor T, Martinez-Yamout M, Dyson HJ, Wright PE (2002). Cooperativity in transcription factor binding to the coactivator CREB-binding protein (CBP) - The mixed lineage leukemia protein (MLL) activation domain binds to an allosteric site on the KIX domain. J Biol Chem.

[39] Ramaswamy K, Forbes L, Minuesa G, Gindin T, Brown F, Kharas MG, et al. Peptidomimetic blockade of MYB in acute myeloid leukemia. Nature Communications. 2018:9:110. 10.1038/s41467-017-02618-6 PMC5760651 29317678

[40] Sánchez-Molina S, Oliva JL, García-Vargas S, Valls E, Rojas JM, Martínez-Balbás MA (2006). The histone acetyltransferases CBP/p300 are degraded in NIH 3T3 cells by activation of Ras signalling pathway. Biochem J.

[41] Liu Y, Wang DL, Chen S, Zhao L, Sun FL (2012). Oncogene Ras/phosphatidylinositol 3-kinase signaling targets histone H3 acetylation at lysine 56. J Biol Chem.

[42] Wei J, Dong S, Bowser RK, Khoo A, Zhang L, Jacko AM, et al. Regulation of the ubiquitylation and deubiquitylation of CREB-binding protein modulates histone acetylation and lung inflammation. Sci Signal. 2017:10:eaak9660. 10.1126/scisignal.aak9660 PMC5863726 28611184

[43] Marambaud P, Wen PH, Dutt A, Shioi J, Takashima A, Siman R (2003). A CBP binding transcriptional repressor produced by the PS1/epsilon-cleavage of N-cadherin is inhibited by PS1 FAD mutations. Cell..

[44] Gao R, Chakraborty A, Geater C, Pradhan S, Gordon KL, Snowden J, et al. Mutant huntingtin impairs PNKP and ATXN3, disrupting DNA repair and transcription. eLife. 2019:8:e42988. 10.7554/eLife.42988 PMC6529219 30994454

[45] Huang WC, Ju TK, Hung MC, Chen CC (2007). Phosphorylation of CBP by IKKalpha promotes cell growth by switching the binding preference of CBP from p53 to NF-kappaB. Mol Cell.

[46] Burma S, Chen BP, Murphy M, Kurimasa A, Chen DJ (2001). ATM phosphorylates histone H2AX in response to DNA double-strand breaks. J Biol Chem.

[47] Waterman DP, Haber JE, Smolka MB (2020). Checkpoint Responses to DNA Double-Strand Breaks. Ann Rev Biochem.

[48] Hoeijmakers JH (2009). DNA damage, aging, and cancer. N Engl J Med.

[49] Shiloh Y, Ziv Y (2013). The ATM protein kinase: regulating the cellular response to genotoxic stress, and more. Nat Rev Mol Cell Biol.

[50] Lee JH, Paull TT (2021). Cellular functions of the protein kinase ATM and their relevance to human disease. Nat Rev Mol Cell Biol.

[51] O’Connor MJ (2015). Targeting the DNA Damage Response in Cancer. Mol Cell.

[52] Hengel SR, Spies MA, Spies M (2017). Small-Molecule Inhibitors Targeting DNA Repair and DNA Repair Deficiency in Research and Cancer Therapy. Cell Chem Biol.

[53] Hickson I, Zhao Y, Richardson CJ, Green SJ, Martin NM, Orr AI (2004). Identification and characterization of a novel and specific inhibitor of the ataxia-telangiectasia mutated kinase ATM. Cancer Res.

[54] Stakyte K, Rotheneder M, Lammens K, Bartho JD, Grädler U, Fuchß T (2021). Molecular basis of human ATM kinase inhibition. Nat Struct Mol Biol.

[55] Estrov Z. The Leukemia Stem Cell. In: Nagarajan L, editor. Acute Myelogenous Leukemia: Genetics, Biology and Therapy. New York, NY: Springer New York; 2010. pp. 1–17.

[56] Lowenberg B (2017). Introduction to the review series on leukemic stem cells. Blood.

[57] Clarke MF (2019). Clinical and Therapeutic Implications of Cancer Stem Cells. N Engl J Med.

[58] Roux B, Vaganay C, Vargas JD, Alexe G, Benaksas C, Pardieu B, et al. Targeting acute myeloid leukemia dependency on VCP-mediated DNA repair through a selective second-generation small-molecule inhibitor. Sci Transl Med. 2021;13:eabg1168. 10.1126/scitranslmed.abg1168 PMC8672851 33790022

[59] Wu D, Zhang J, Jun Y, Liu L, Huang C, Wang W (2024). The emerging role of DOT1L in cell proliferation and differentiation: Friend or foe. Histol Histopathol.

[60] He ZX, Wei BF, Zhang X, Gong YP, Ma LY, Zhao W (2021). Current development of CBP/p300 inhibitors in the last decade. Eur J Med Chem.

[61] Barlow C, Hirotsune S, Paylor R, Liyanage M, Eckhaus M, Collins F (1996). Atm-deficient mice: a paradigm of ataxia telangiectasia. Cell.

[62] Stewart SA, Dykxhoorn DM, Palliser D, Mizuno H, Yu EY, An DS (2003). Lentivirus-delivered stable gene silencing by RNAi in primary cells. RNA.

[63] Naviaux RK, Costanzi E, Haas M, Verma IM (1996). The pCL vector system: rapid production of helper-free, high-titer, recombinant retroviruses. J Virol.

[64] Saito Y, Chapple RH, Lin A, Kitano A, Nakada D (2015). AMPK Protects Leukemia-Initiating Cells in Myeloid Leukemias from Metabolic Stress in the Bone Marrow. Cell Stem Cell.

[65] Okada Y, Feng Q, Lin Y, Jiang Q, Li Y, Coffield VM (2005). hDOT1L links histone methylation to leukemogenesis. Cell.

[66] Li Z, Wang F, Tian X, Long J, Ling B, Zhang W (2021). HCK maintains the self-renewal of leukaemia stem cells via CDK6 in AML. J Exp Clin Cancer Res.

[67] Schmittgen TD, Livak KJ (2008). Analyzing real-time PCR data by the comparative C(T) method. Nat Protoc.

[68] Kim D, Langmead B, Salzberg SL (2015). HISAT: a fast spliced aligner with low memory requirements. Nat Methods.

[69] Langmead B, Salzberg SL (2012). Fast gapped-read alignment with Bowtie 2. Nat Methods.

[70] Li B, Dewey CN (2011). RSEM: accurate transcript quantification from RNA-Seq data with or without a reference genome. BMC Bioinformatics.

[71] Love MI, Huber W, Anders S (2014). Moderated estimation of fold change and dispersion for RNA-seq data with DESeq2. Genome Biol.

[72] Huang da W, Sherman BT, Lempicki RA (2009). Systematic and integrative analysis of large gene lists using DAVID bioinformatics resources. Nat Protoc.

[73] Sherman BT, Hao M, Qiu J, Jiao X, Baseler MW, Lane HC (2022). DAVID: a web server for functional enrichment analysis and functional annotation of gene lists (2021 update). Nucleic Acids Res.

[74] Bagger FO, Sasivarevic D, Sohi SH, Laursen LG, Pundhir S, Sønderby CK (2016). BloodSpot: a database of gene expression profiles and transcriptional programs for healthy and malignant haematopoiesis. Nucleic Acids Res.

[75] Tang Z, Kang B, Li C, Chen T, Zhang Z (2019). GEPIA2: an enhanced web server for large-scale expression profiling and interactive analysis. Nucleic Acids Res.

