# Supplementary material for: The DNA damage-independent ATM signalling maintains CBP/DOT1L axis in MLL rearranged acute myeloid leukaemia
Source: Oncogene. 2024 Apr 26;43(25):1900–16. doi: 10.1038/s41388-024-02998-2 (PMC11178498; doi:10.1038/s41388-024-02998-2)
Supplement: Supplementary file 1 — Supplementary figures and legends [file 41388_2024_2998_MOESM1_ESM.pdf]

Figure S1

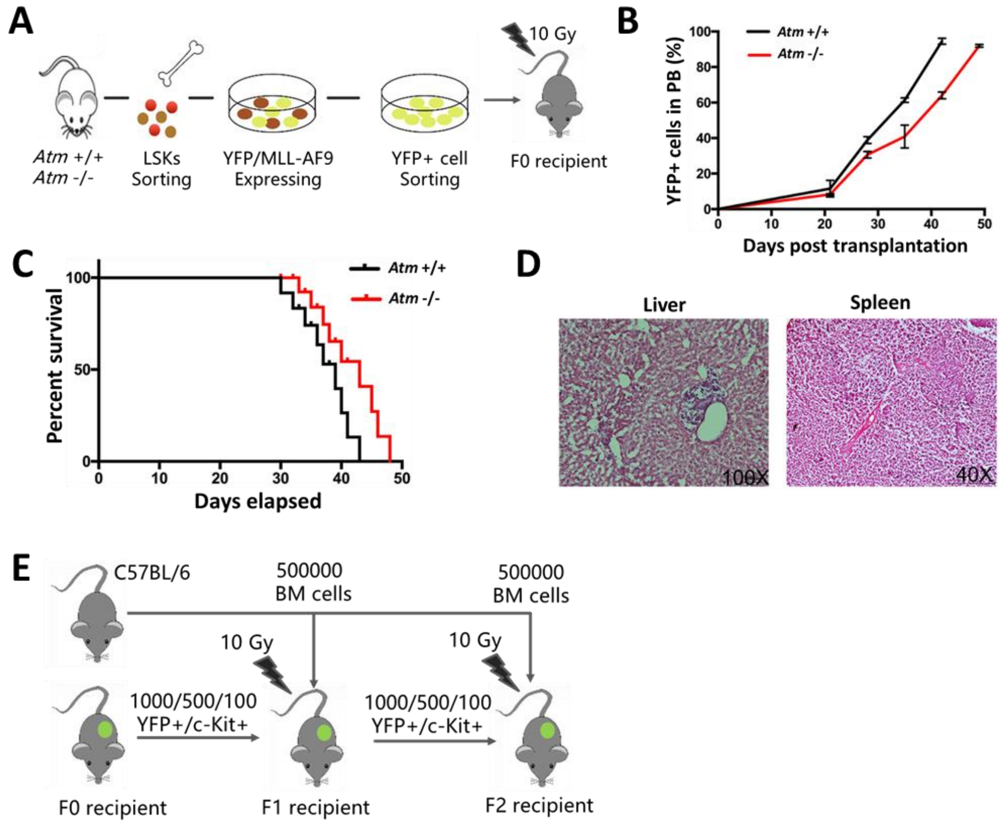

Figure S1. ***Atm*** **is required for the long-term maintenance of LSCs in MLL-AF9-AML mice.**

A. Schematic interpretation of the generation of murine LSCs. LSKs ($Lin^-/Sca\text{-}1^+/c\text{-}kit^+$) from $Atm^{+/+}$ or $Atm^{-/-}$ mice bone marrow were sorted and then ectopically expressed YFP/*MLL-AF9*. The YFP^+ cells were LSKs expressing MLL-AF9. Next, the YFP^+ cells from either $Atm^{+/+}$ or $Atm^{-/-}$ mice were collected and injected into lethally irradiated wild-type C57BL/6 mice to generate $Atm^{+/+}$ or $Atm^{-/-}$ F0 recipients. **B.** The YFP^+ cells in peripheral blood (PB) of $Atm^{+/+}$ and $Atm^{-/-}$ F0 recipients were analysed weekly by FACS 3 weeks post-transplantation. n=10 mice for each group. **C.** The overall survival of $Atm^{+/+}$ and $Atm^{-/-}$ F0 recipients were analysed by Kaplan-Meier plotting. n=10 mice for each group. **D.** Representative images showed the hematoxylin-eosin staining of the liver and spleen from F0 recipient mice 6 weeks after transplantation. **E.**

Schematic interpretation of the induction of mouse AML with murine LSC transplantation. LSCs (YFP^+/c-kit^+) from F0 recipients were collected. Next, 1,000/500/100 LSCs, in combination with 50,000 bone marrow (BM) cells from wild-type C57BL/6 mice, were injected into lethally irradiated wild-type C57BL/6 mice to generate F1. The isolated LSCs from F1 were then used to generate F2 recipients.

Figure S2

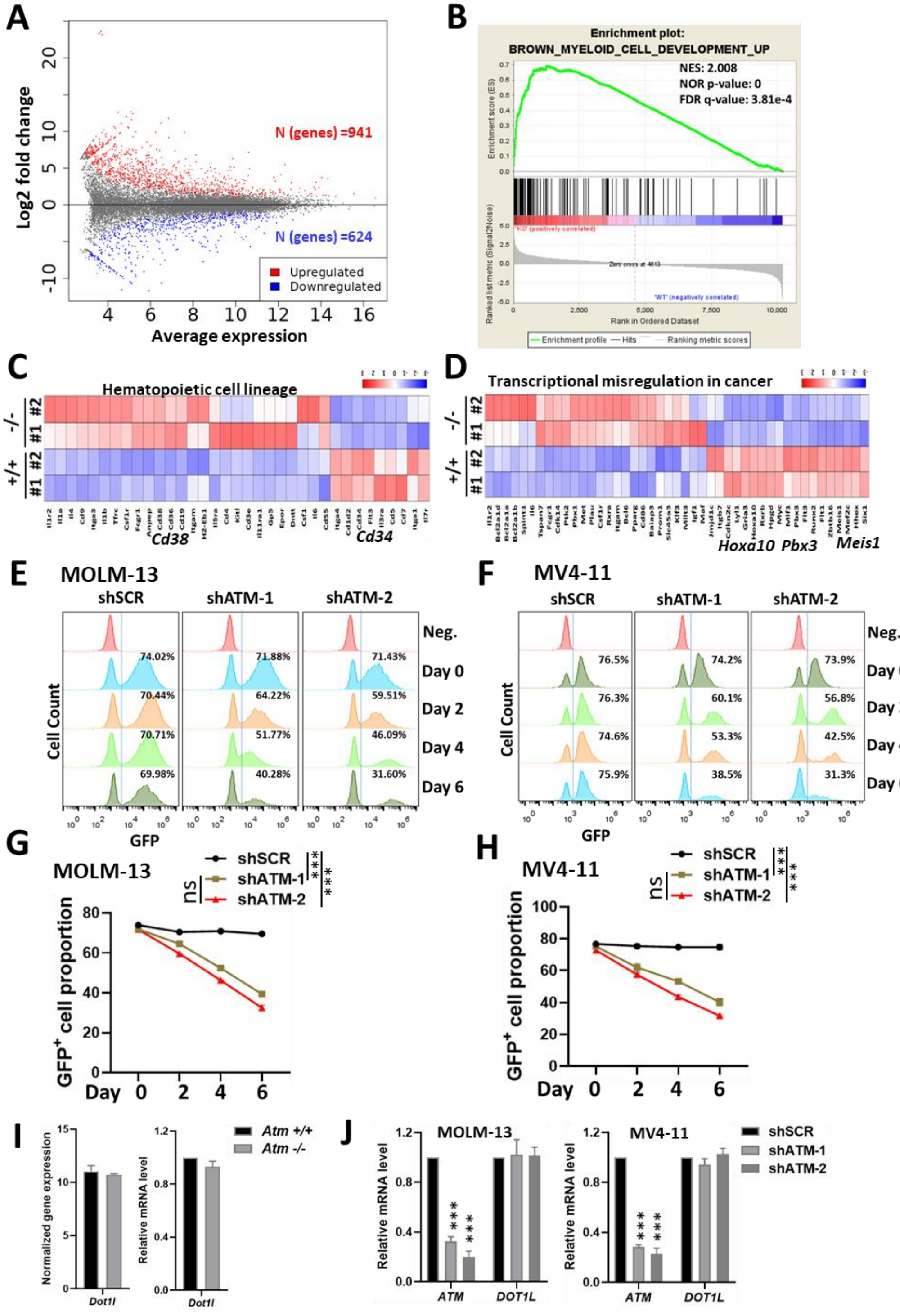

Figure S2. ATM deficiency downregulates the DOT1L/H3K79me pathway in MLLr-AML.

A. The MA plot displayed the differentially expressed genes between LSCs from *Atm*$^{+/+}$ and *Atm*$^{-}$

$^{/-}$ F2 recipients. **B.** GSEA (Gene Set Enrichment Analysis) analysis showed the gene expression differences between LSCs from *Atm*$^{+/+}$ and *Atm*$^{-/-}$ F2 recipients were enriched in BROWN_MYELOID_CELL_DEVELOPMENT_UP. **C, D.** Heatmap showed the differentially expressed genes related to hematopoietic cell lineage **(C)** and transcriptional misregulation in cancer **(D)**. **E, F.** The cells were transduced with lentivirus expressing shRNA and green fluorescent protein (GFP). The histograms showed the GFP^{+} cell proportion change during culture for MOLM-13 **(E)** and MV4-11 **(F)** cells transduced with shRNA expressing lentivirus. **G, H.** The cells were subjected to flow cytometry to analyse the GFP^{+} cell proportion in MOLM-13 **(G)** and MV4-11 **(H)** cells. The analysis was performed every two days during the culture. Three replicates for each time point of different cells were used for linear regression analysis. The difference in slopes between every two groups was compared. *** $P<0.001$. Three independent experiments were performed. **I.** The normalised gene expression of *Dot1l* from RNA-Seq of LSCs from *Atm*$^{+/+}$ and *Atm*$^{-/-}$ F2 recipients were analysed (left). The relative mRNA level of *Dot1l* in LSCs from *Atm*$^{+/+}$ and *Atm*$^{-/-}$ F2 recipients were quantified with quantitative RT-PCR (right). Three replicates were used for quantification (mean ± SD). **J.** The relative mRNA levels of *ATM* and *DOT1L* in control (shSCR) and *ATM* knocked down (shATM) MOLM-13 and MV4-11 cells were quantified with quantitative RT-PCR. Three replicates for each gene were used for quantification (mean ± SD). *** $P<0.001$. Student's t-test.

Figure S3

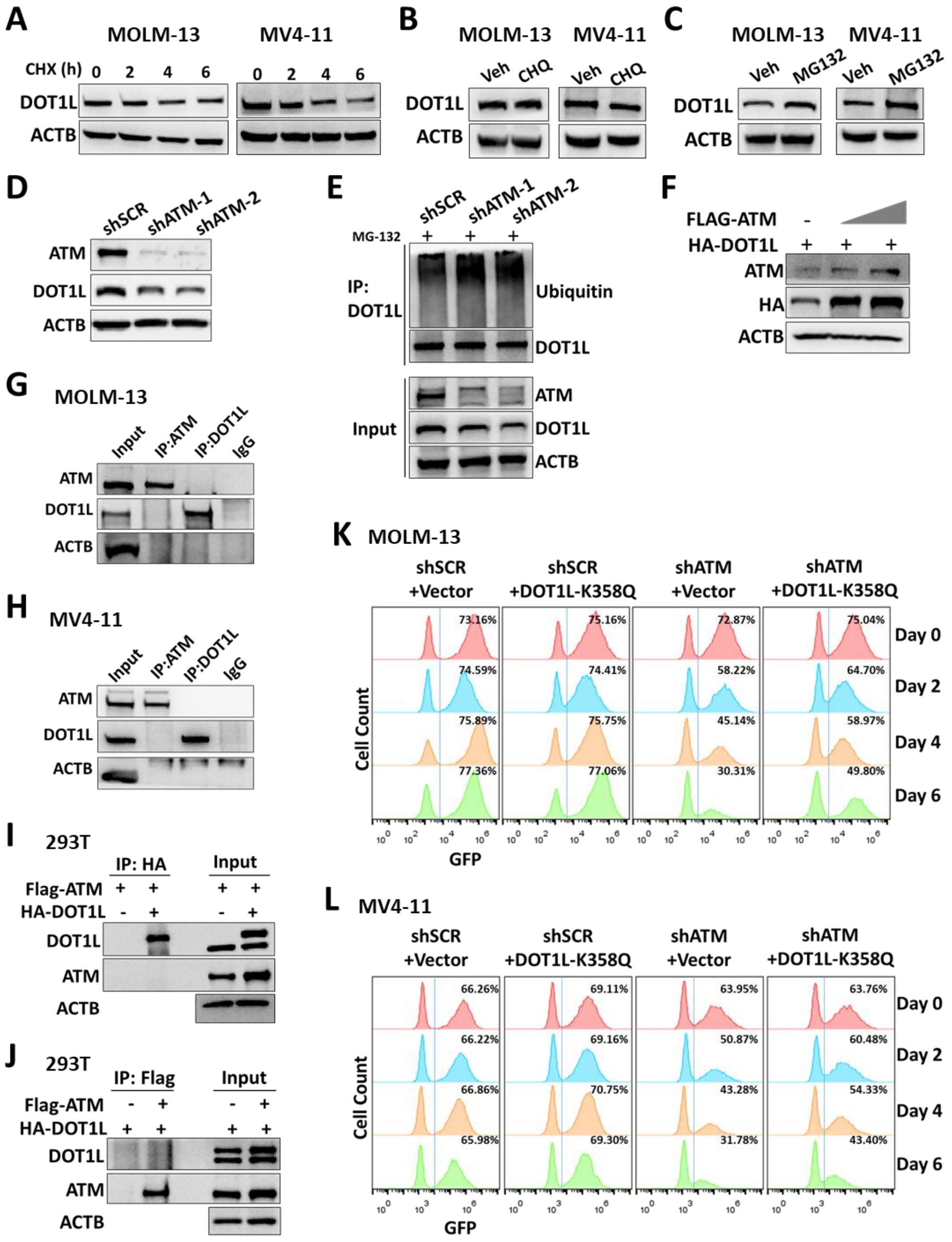

Figure S3. ATM protects DOT1L protein from ubiquitination-mediated degradation. A. MOLM-13 and MV4-11 cells were incubated with 40 μg/ml cycloheximide (CHX) for the indicated time. The total lysates of the cells were then subjected to western blotting with the indicated antibodies. **B.** MOLM-13 and MV4-11 cells were incubated with DMSO (Vehicle, Veh)

or 50 μM chloroquine (CHQ) for 24 h. The total lysates of the cells were then subjected to western blotting with the indicated antibodies. **C.** MOLM-13 and MV4-11 cells were incubated with 40 μg/ml CHX and DMSO (Vehicle, Veh) or 25 μM MG132 for 6 h. The total lysates of the cells were then subjected to western blotting with the indicated antibodies. **D.** The total lysates of the control (shSCR) and *ATM* knocked down (shATM) 293T cells were subjected to western blotting with the indicated antibodies. **E.** The control (shSCR) and *ATM* knocked down (shATM) 293T cells were treated with 25 μM MG132 for 20 h. The cell lysates were subjected to IP with DOT1L antibodies. The Input and IP samples were then detected by western blotting with the indicated antibodies. **F.** 293T cells were co-transfected with HA-DOT1L and control vector (Flag-Vec) or Flag-ATM. The cell lysates were then subjected to western blotting with the indicated antibodies. **G, H.** MOLM-13 **(G)** and MV4-11 **(H)** cell lysates were subjected to IP with ATM, DOT1L antibodies, and control IgG. The Input and IP samples were then detected by western blotting with the indicated antibodies. **I.** Flag-ATM with or without HA-DOT1L were co-transfected into 293T cells. The cell lysates were then subjected to IP with HA antibodies. The Input and IP samples were then detected by western blotting with the indicated antibodies.
J. HA-DOT1L with or without Flag-ATM were co-transfected into 293T cells. The cell lysates were then subjected to IP with Flag antibodies. The Input and IP samples were then detected by western blotting with the indicated antibodies. **K, L.** The histograms showed the GFP^+ cell proportion change during culture for MOLM-13 **(K)** and MV4-11 **(L)** cells.

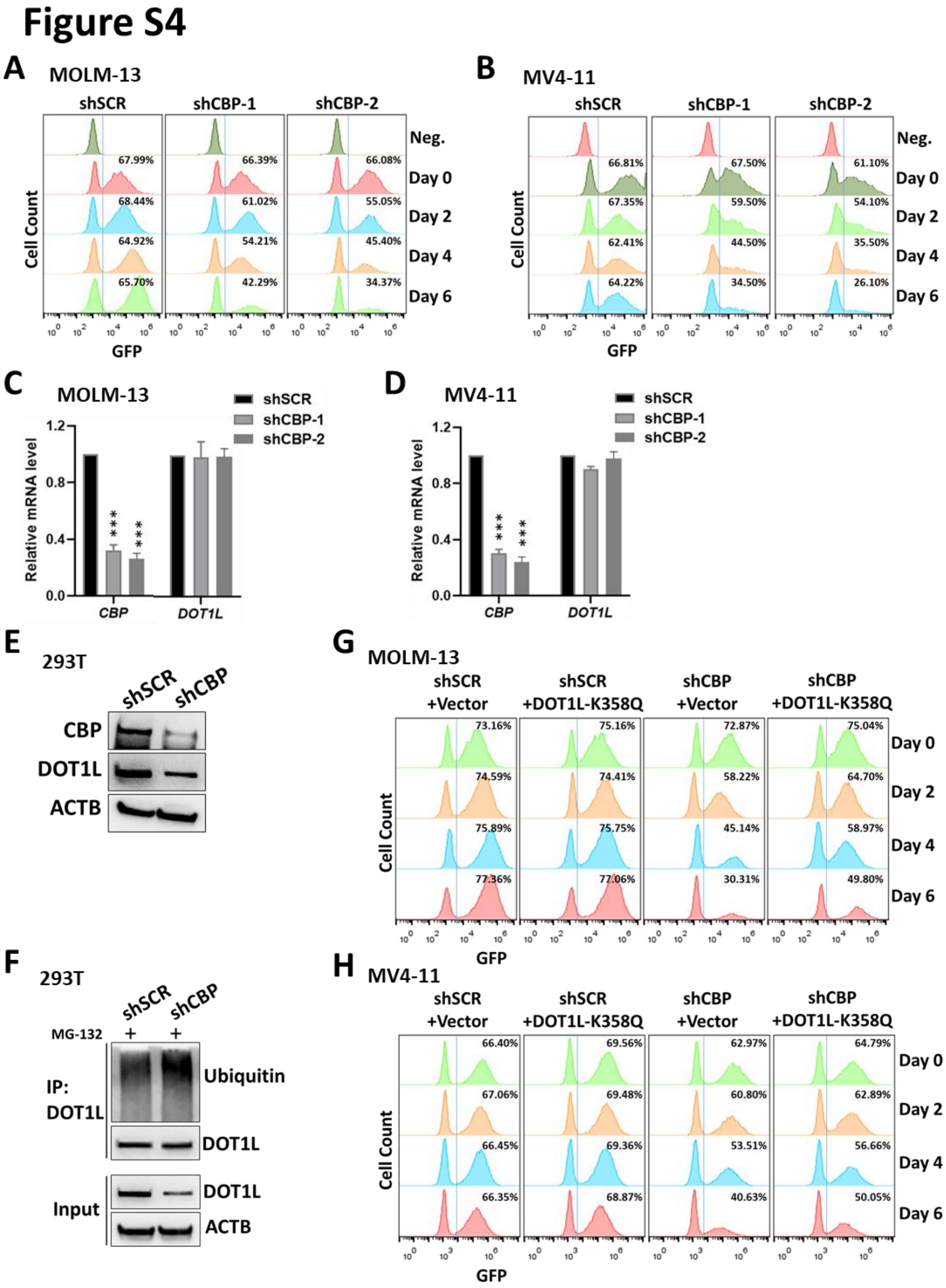

Figure S4. CBP-mediated DOT1L-K358 acetylation confers DOT1L stability in *MLLr*-AML. A, B. The histograms showed the GFP^{+} cell proportion change during culture for MOLM-13 **(A)** and MV4-11 **(B)** cells. **C, D.** The relative mRNA levels of *CBP* and *DOT1L* in control (shSCR) and *CBP* knocked down (shATM), MOLM-13 **(C),** and MV4-11 **(D)** cells were

quantified with quantitative RT-PCR. Three replicates for each gene were used for quantification (mean ± SD). *** $P<0.001$. Student's t-test. **E.** Control (shSCR) and *CBP* knocked down (shCBP) 293T cell lysates were subjected to western blotting with the indicated antibodies. **F.** Control (shSCR) and *CBP* knocked down (shCBP) 293T cells were treated with 25 μM MG132 for 20 h. The cell lysates were subjected to immunoprecipitation (IP) with DOT1L antibodies. The Input and IP samples were then detected by western blotting with the indicated antibodies. **G, H.** The histograms showed the GFP^+ cell proportion change during culture for MOLM-13 **(G)** and MV4-11 **(H)** cells.

Figure S5

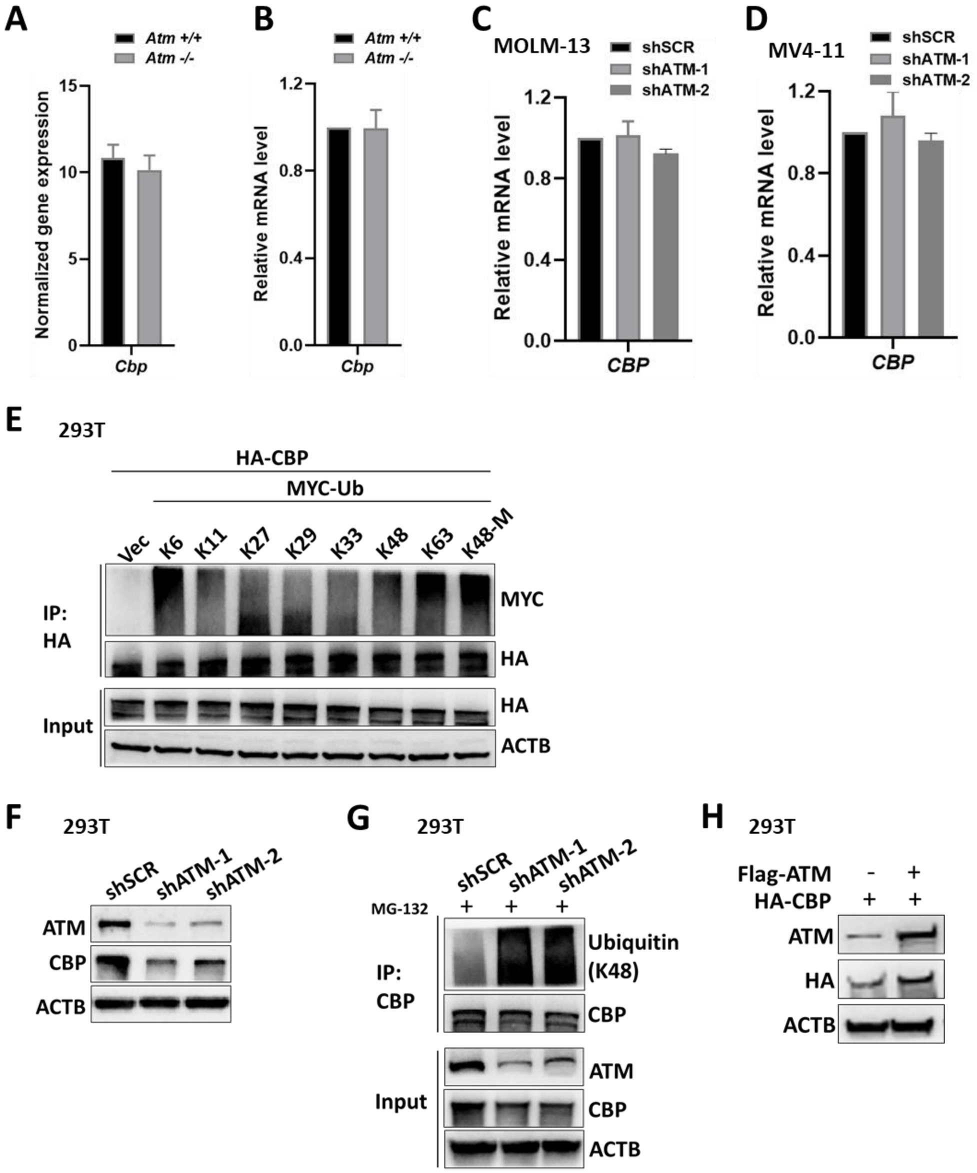

Figure S5. ATM confers CBP stability by preventing its ubiquitination-mediated degradation. A. The normalised gene expression of *Cbp* from RNA-Seq of LSCs from *Atm*$^{+/+}$ and *Atm*$^{-/-}$ F2 recipients were analysed. **B.** The relative mRNA level of *Cbp* in LSCs from *Atm*$^{+/+}$ and *Atm*$^{-/-}$ F2 recipients were quantified with quantitative RT-PCR. Three replicates were used for quantification (mean ± SD). **C, D.** The relative mRNA levels of *CBP* in control (shSCR) and

ATM knocked down (shATM) MOLM-13 **(C)** and MV4-11 **(D)** cells were quantified with quantitative RT-PCR. Three replicates for each gene were used for quantification (mean ± SD). **E.** HA-CBP with different MYC-tagged ubiquitin (MYC-Ub) variants were co-transfected into 293T cells. For MYC-Ub variants, all lysines (K) in ubiquitin were mutated to alanines except the labelled one. 36 h post-transfection, the K48-MYC-Ub cells were treated with 25 μM MG132 for 20 h (K48-M). The cell lysates were subjected to IP with HA antibodies. The Input and IP samples were then detected by western blotting with the indicated antibodies. **F.** Control (shSCR) and *ATM* knocked down (shATM) 293T cell lysates were subjected to western blotting with the indicated antibodies. **G.** Control (shSCR) and *ATM* knocked down (shATM) 293T cells were treated with 25 μM MG132 for 20 h. The cell lysates were subjected to IP with CBP antibodies. The Input and IP samples were then detected by western blotting with the indicated antibodies. **H.** HA-CBP with or without Flag-ATM were co-transfected into 293T cells. 72 h later, the cells were collected, and the cell lysates were then subjected to western blotting with the indicated antibodies.

Figure S6

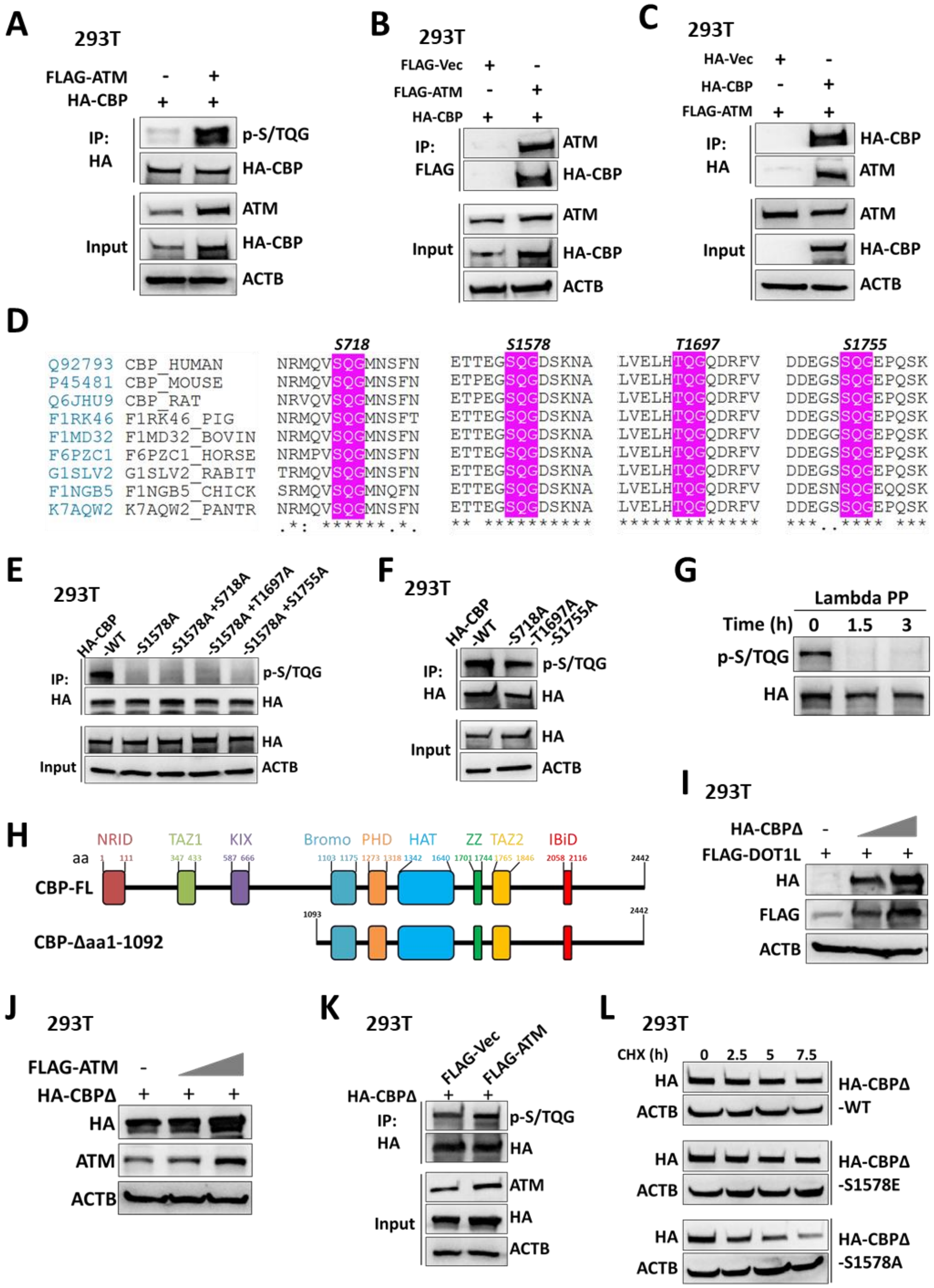

Figure S6. S1578 phosphorylation mediated by ATM confers the stability of CBP. A. HA-CBP with or without Flag-ATM were co-transfected into 293T cells. 72 h later, the cells were collected, and the cell lysates were then subjected to IP with HA antibodies. The Input and IP

samples were then detected by western blotting with the indicated antibodies. **B.** HA-CBP with or without Flag-ATM were co-transfected into 293T cells. The cell lysates were then subjected to IP with FLAG antibodies. The Input and IP samples were then detected by western blotting with the indicated antibodies. **C.** Flag-ATM with or without HA-CBP were co-transfected into 293T cells. The cell lysates were then subjected to IP with HA antibodies. The Input and IP samples were then detected by western blotting with the indicated antibodies. **D.** The S/TQG sites (S718, S1578, T1697, and S1755) in CBP protein across humans, monkeys, mice, rats, pigs, bovines, horses, rabbits, and chickens were analysed in the online UniProt database. **E.** The WT, S1578A, S1578A/S718A, S1578A/T1697A, and S578A/S1755A HA-CBP were transfected into 293T cells. 72 h later, the cell lysates were subjected to IP with HA antibodies. The Input and IP samples were then detected by western blotting with the indicated antibodies. **F.** The WT and S718A/T1697A/S1755A HA-CBP were transfected into 293T cells. 72 h later, the cell lysates were subjected to IP with HA antibodies. The Input and IP samples were then detected by western blotting with the indicated antibodies. **G.** The purified HA-CBP was subjected to *in vitro* dephosphorylation with Lambda PP (protein phosphatase) at 30°C for the indicated time. The reactions were detected by western blotting with the indicated antibodies. **H.** Image showed the full-length CBP (CBP-FL) and the truncated one (CBP-Δ). Amino acids 1 to 1092 were deleted in CBP-Δ, compared to CBP-FL. **I.** FLAG-DOT1L with or without HA-CBPΔ were co-transfected into 293T cells. 72 h later, the cells were collected, and the cell lysates were then subjected to western blotting with the indicated antibodies. **J.** HA-CBPΔ with or without FLAG-ATM were co-transfected into 293T cells. 72 h later, the cells were collected, and the cell lysates were then subjected to western blotting with the indicated antibodies. **K.** HA-CBPΔ with or without FLAG-ATM were co-transfected into 293T cells. 72 h later, the cell lysates were

subjected to IP with HA antibodies. The Input and IP samples were then detected by western blotting with the indicated antibodies. **L.** The 293T cells were transduced with lentivirus expressing wild-type (WT) or mutant HA-CBPΔ (S1578E, S1578A). The cells were incubated with 40 μg/ml CHX for the indicated time. The total lysates of the cells were then subjected to western blotting with the indicated antibodies.

Figure S7

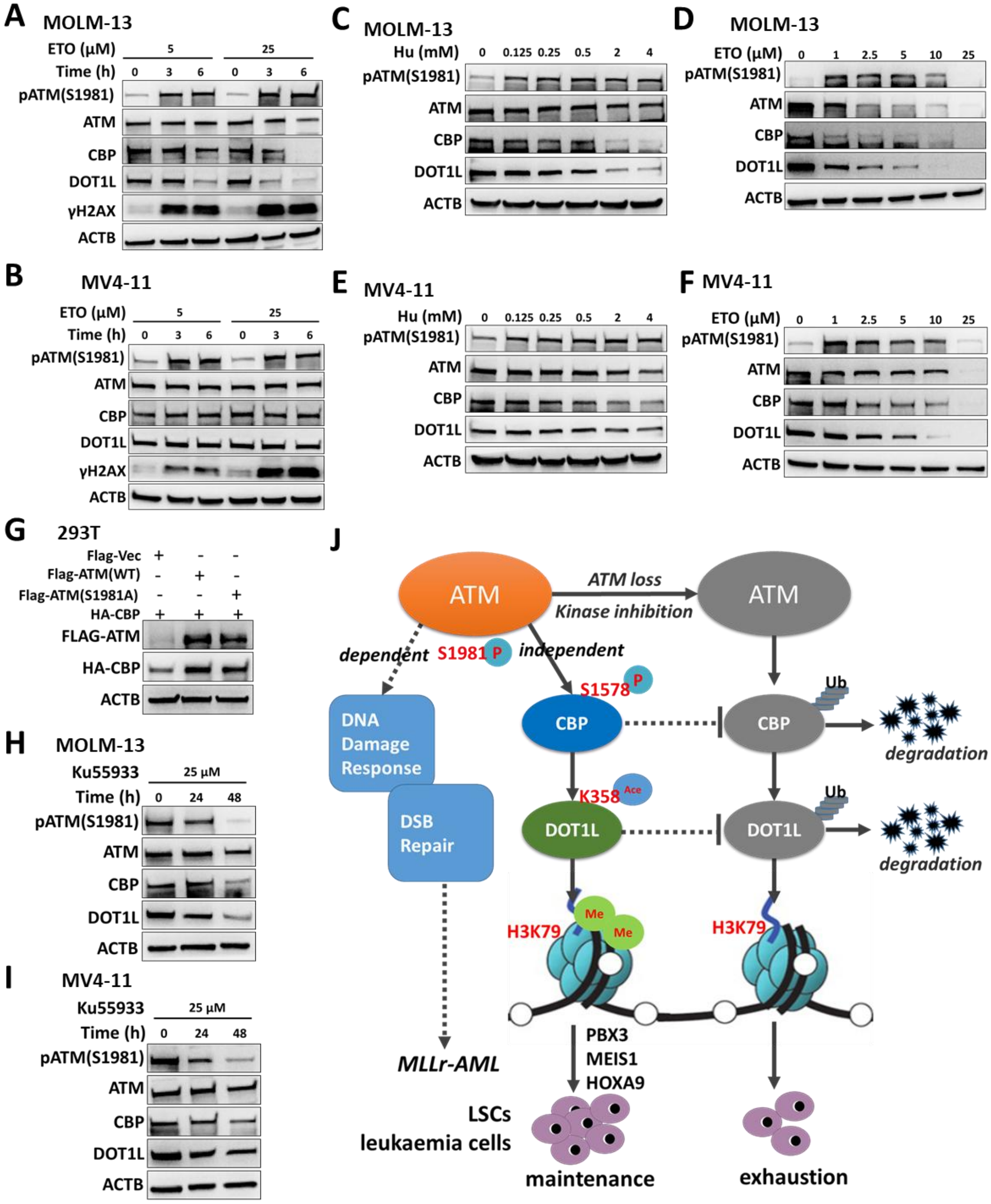

Figure S7. The kinase activity of ATM for the regulation of CBP and DOT1L is DNA damage independent. A, B. MOLM-13 (A) and MV4-11 (B) cells were treated with etoposide (ETO, 5 μM or 25 μM) for the indicated time. The lysates were extracted and detected by

western blotting with the indicated antibodies. **C-F.** MOLM-13 (C, D) and MV4-11 (E, F) cells were treated with different doses of hydroxyurea (Hu) or etoposide (ETO) for 24 h. The lysates were extracted and detected by western blotting with the indicated antibodies. **G.** HA-CBP with FLAG-Vec, FLAG-ATM (WT), or FLAG-ATM(S1981A) were co-transfected into 293T cells. 72 h later, the cells were collected, and the cell lysates were then subjected to western blotting with the indicated antibodies. **H, I.** MOLM-13 (H) and MV4-11 (I) cells were treated with 25 μM Ku55933 for the indicated time. The cell lysates were then subjected to western blotting with the indicated antibodies. **J.** The proposed role of ATM protein in *MLLr-AML*. Classically, the phosphorylation of ATM(S1981) is required for DNA damage response and the repair of DNA double-strand breaks in *MLLr-AML* cells. Besides, an ATM(S1981) independent pathway exists. ATM phosphorylates CBP(S1578) to maintain its stability; the stabilised CBP then functions to DOT1L acetylation and the subsequent H3K79 methylation to maintain leukaemia stem cells and leukaemia cells in *MLLr-AML*. The loss of the *ATM* gene or the kinase inhibition of the ATM protein promotes the proteasomal degradation of CBP protein and the breakage of DOT1L-H3K79me signalling, ultimately leading to the exhaustion of *MLLr-AML* cells.
